# Using syndrome mining with the Health and Retirement Study to identify the deadliest and least deadly frailty syndromes

**DOI:** 10.1038/s41598-020-60869-8

**Published:** 2020-04-08

**Authors:** Yi-Sheng Chao, Chao-Jung Wu, Hsing-Chien Wu, Hui-Ting Hsu, Lien-Cheng Tsao, Yen-Po Cheng, Yi-Chun Lai, Wei-Chih Chen

**Affiliations:** 1 Independent researcher, Montréal, Canada; 2 Département d’informatique, Université du Québec à Montréal, Montréal, Canada; 3 Taipei Hospital, Ministry of Health and Welfare, Taipei, Taiwan; 4 Changhua Christian Hospital, Changhua, Taiwan; 5 Division of Chest Medicine, Department of Internal Medicine, National Yang-Ming University Hospital, Yi-Lan, Taiwan; 6 Department of Chest Medicine, Taipei Veterans General Hospital, Taipei, Taiwan; 7 Faculty of Medicine and Institute of Emergency and Critical Care Medicine, School of Medicine, National Yang-Ming University, Taipei, Taiwan

**Keywords:** Geriatrics, Epidemiology

## Abstract

Syndromes are defined with signs or symptoms that occur together and represent conditions. We use a data-driven approach to identify the deadliest and most death-averse frailty syndromes based on frailty symptoms. A list of 72 frailty symptoms was retrieved based on three frailty indices. We used data from the Health and Retirement Study (HRS), a longitudinal study following Americans aged 50 years and over. Principal component (PC)-based syndromes were derived based on a principal component analysis of the symptoms. Equal-weight 4-item syndromes were the sum of any four symptoms. Discrete-time survival analysis was conducted to compare the predictive power of derived syndromes on mortality. Deadly syndromes were those that significantly predicted mortality with positive regression coefficients and death-averse ones with negative coefficients. There were 2,797 of 5,041 PC-based and 964,774 of 971,635 equal-weight 4-item syndromes significantly associated with mortality. The input symptoms with the largest regression coefficients could be summed with three other input variables with small regression coefficients to constitute the leading deadliest and the most death-averse 4-item equal-weight syndromes. In addition to chance alone, input symptoms’ variances and the regression coefficients or p values regarding mortality prediction are associated with the identification of significant syndromes.

## Introduction

Syndromes can be defined by signs or symptoms that occur together and are representative of certain conditions^1^. Syndromes can represent conditions rooted in genetic mutations, pathological changes or concurrent symptoms^2^. For example, Down syndrome is a genetic disorder characterized by facial features and intellectual disability^2^. Syndromes can be identified through theoretic frameworks, expert opinions, or empirical evidence. Among candidate patients, an identified group of symptoms can constitute a syndrome^3–5^. In some cases, the underlying causes, such as genetic abnormalities and pathological processes, can be illustrated^6,7^.

However, definitions of syndromes can vary among health organizations. For example, metabolic syndrome has been described with at least two sets of different criteria: one proposed by the World Health Organization and another by the National Cholesterol Education Program in the United States (US)^8^. The definitions of metabolic syndrome are also under intense criticism for the lack of clinical significance for several health outcomes^9,10^.

Frailty syndromes have been defined and measured differently^11–18^. Different criteria and collections of frailty symptoms have been tested in the elderly^11,12^. New indices are frequently proposed by separate groups of researchers to provide alternative measures of frailty^18^. However, there is emerging evidence indicating that even syndromes that are well defined based on a collection of related clinical symptoms or are supported by theories and empirical research may fail to represent the conditions the syndromes aim to introduce^12^. It may not be easy to reach a consensus about how syndromes can be defined, especially those not rooted in genetic or pathological findings^19^.

Conceptually, frailty syndromes are similar to composite measures or indices that are sums of multiple input variables with equal or unequal weights^20^. Given how differently they are measured and the distinctive theories that inspired them, it is surprising that most frailty syndromes are significantly associated with major health outcomes, especially mortality. One reason is that significant health outcomes may be more likely to be published^21^. Alternatively, there are numerous candidate syndromes to screen, test, and publish. Recent findings in index mining suggest that syndromes can be searched systematically using large data sets and pre-specified rules^20^. For example, there are 72 frailty symptoms identified to form frailty syndromes in the Health and Retirement Study (HRS), and a large number of possible combinations are available^20,22^. Facing this large number of candidate syndromes, there are no well-established criteria to select clinically meaningful syndromes regardless of its statistical significance^18^. The underlying causes associated with new statistically significant frailty syndromes with important outcomes have not been identified. It is necessary to identify the factors contributing to statistical significances of frailty syndromes before assessing the importance of statistical significances in frailty syndromes. Then, a set of criteria for the selection of clinical meaningful syndromes could be developed. This study aims to identify the factors related to the statistical significances of newly generated syndromes, taking frailty syndromes as an example. The characteristics of the newly identified frailty syndromes with the largest magnitudes of regression coefficients or the least p values are also discussed.

## Results

In 2004, there were 11,025 Health and Retirement Study (HRS) participants interviewed. 57.58% of the HRS participants were female with a mean age of 74.95 years [95% confidence interval (CI) = 74.87 to 75.02]. The mean follow-up time for all participants was 7.46 years. The mean follow-up times for deceased and surviving participants were 4.93 and 9.51 years respectively. Survival curves and other details are published elsewhere^23^.

### Number of significant syndromes

**Figure 1:**
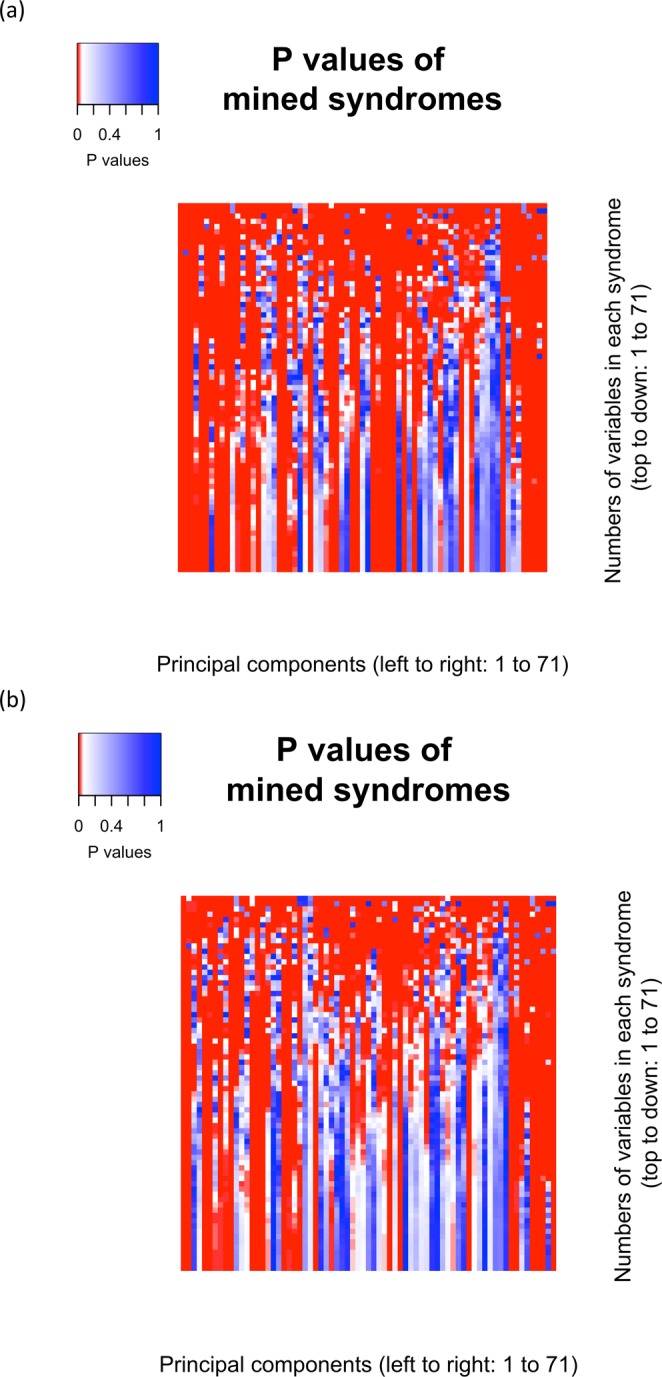
Principal component-based syndrome p values for the prediction of mortality. (**a**) Principal component-based syndromes without controlling for sex, race/ethnicity, education per capita income, and per capita wealth. (**b**) Principal component-based syndromes with sex, race/ethnicity, education per capita income, and per capita wealth controlled.

There were 5,041 PC-based syndromes and 971,635 equal-weight 4-item syndromes mined. Mortality prediction p values ranged from approximately zero to one, (adjusted for multiple comparisons or not) observed for both types of syndromes predicting mortality prediction, controlling for sex, race/ethnicity, education, per capita income, and per capita wealth. Principal component (PC)-based syndrome p values are shown in Fig. 1 and Appendix 1 (adjusted for multiple comparisons). There were fewer syndromes significantly associated with mortality after controlling for demographic characteristics (2,797 PC-based and 964,774 4-item syndromes; 55.5% and 99.3% for each type, respectively). After adjusting for multiple comparisons, there remained 1,455 and 964,694 significant syndromes respectively (28.9% and 99.3% respectively).

**Figure 2:**
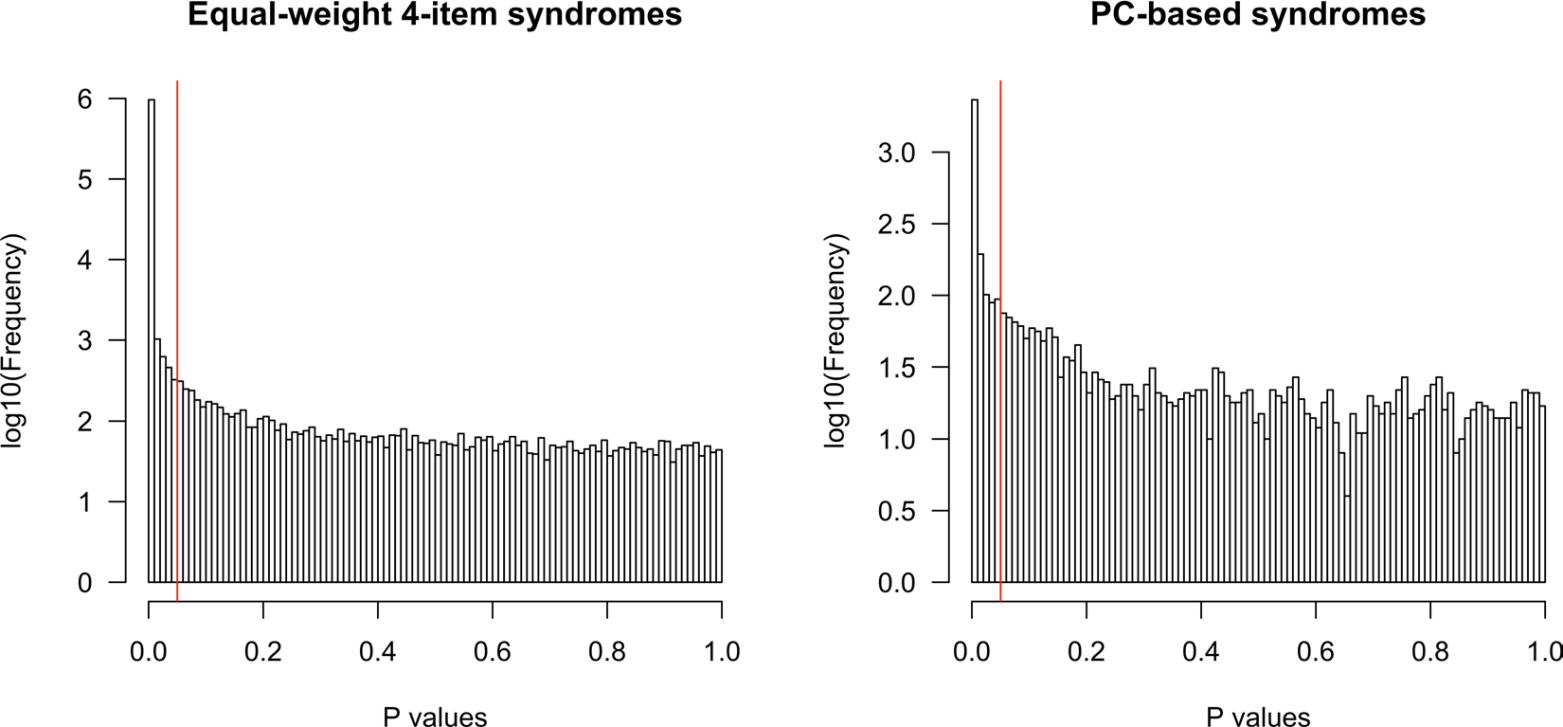
Distribution of the syndromes based on p values. P values derived after accounting for sex, race/ethnicity, education, per capita income, and per capita wealth. Red line = 0.05.

The distributions of p values derived from mortality prediction for PC-based and equal-weight syndromes are shown in Fig. 2 and Table 1. The vertical axis, frequencies, in Fig. 2 is in log scales. There were 1,271 PC-based and 960,446 equal-weight syndromes that were significantly and positively associated with mortality (i.e. deadly, 25.2% and 98.8% of each type, respectively). The other 1,526 PC-based and 4,328 equal-weight syndromes were significantly and negatively associated with mortality (i.e. death-averse, 30.3% and 0.4% of each type respectively).

**Table 1:** Summary of the mined syndromes based on principal components analysis and equal weighting.

	PC-based syndromes	Equal-weight 4-item syndromes
Number of input variables	71	71
Ineligible input variables	Seizures, generalized (variable name = r7seizure)	Mild to severe cognitive impairment on performance-based measure or according to proxy and interviewer rating (variable name = r7frail1_3)
Number of syndromes mined	5,041	971,635
Ranges of p values	0 to 1	0 to 1
Number of significant syndromes	2,797	964,774
Proportion of significant syndromes	0.555	0.993
Number of significant deadly syndromes (positive coefficients)	1,271	960,446
Proportion of deadly syndromes relative to significant ones	0.252	0.988
Number of significant death-averse syndromes (negative coefficients)	1,526	4,328
Proportion of death-averse syndromes relative to significant syndromes	0.303	0.004

P values and significance are derived from a discrete-time event history survival analysis predicting mortality among the Health and Retirement Study participants, while adjusting for sex, race/ethnicity, education, per capita income and per capita wealth.

### The role of input symptoms

**Figure 3:**
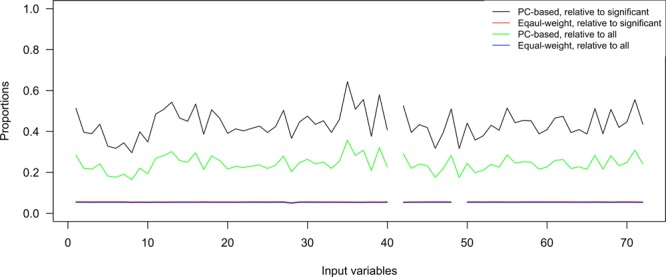
The proportions of the input variables appearing in the syndromes. The numbers on the horizontal axis represent the serial numbers of the input symptoms or conditions in Appendix 1. Input variables with zero variances or missing values were not eligible for PC-based and equal-weight syndromes respectively.

The probabilities of the input symptoms constituting significant syndromes are displayed in Fig. 3. There was one variable with zero variance ineligible for PC-based syndromes, “generalized seizure” (variable name = r7seizure). Two variables with missing values were not eligible for equal-weight syndromes: “generalized seizure” and “mild to severe cognitive impairment on performance-based measure or according to proxy and interviewer rating” (r7seizure and r7frail1_3). The mean probabilities of constituting all PC-based and equal-weight syndromes were 24.2% (95% CI = 23.4% to 25.0%) and 5.3% (95% CI = 5.3% to 5.3%) for the input variables respectively. The mean probabilities of constituting significant PC-based and equal-weight syndromes were 43.6% (95% CI = 42.0% to 45.2%) and 5.6% (95% CI = 5.6% to 5.6%) respectively.

### Most significant syndromes

There were 59 PC-based syndromes with p values approximately zero (Table 2). They were all generated from input variables weighted by the loadings of the first PC (PC1). The 70 leading PC-based syndromes in terms of p values were positively associated with mortality, i.e. deadly syndromes. The 71^st^ to 78^th^ PC-based syndromes were negatively associated with mortality with p values close to zero, i.e. death-averse syndromes. The absolute values of the coefficients of 71^st^ to 77^th^ PC-based syndromes could be extremely large because the mean values of the PC-based syndromes based on the weighting schemes according to PCA loadings were very small. Therefore, the magnitude of the regression coefficients for PC-based syndromes were not used to rank the importance of the PC-based syndromes. Table 3 lists the leading 4-item equal-weight syndromes positively or negatively associated with mortality. The mean values were all positive values. For the positive or negative associations with mortality, the regression coefficients could be positive or negative respectively.

**Table 2:** The leading principal component-based syndromes for mortality prediction in terms of p values.

Ranking	Regression coefficients	Log(p)	Mean	SD	PC	Numbers of variables	Variables
1 to 59	0.19 to 0.46	Log(0)	<0.001	1.26 to 3.28	1	9, 11, 15 to 71	r7mobila, r7bathcat, r7bath, …
60 to 70	0.33 to 1.34	−322 to −223	<0.001	0.36 to 1.77	1	2 to 8, 10, 12, to 14	r7mobila, r7bathcat, r7bath, …
71 to 77	−2.87 to −3.04 ×10^14^	−206 to −222	<0.001	1.62 to 1.75 ×10^−15^	68	39 to 45	r7sleepr, r7sleeprcat, r7diabs, …
78	−0.89	−203.82	<0.001	0.53	40	1	r7mobila
79	2.16	−203.82	<0.001	0.22	1	1	r7mobila
80	−2.93 ×10^14^	−202.39	<0.001	<0.001	68	38	r7sleepr, r7sleeprcat, r7diabs, …

Positive regression coefficients suggesting the syndrome positively correlated with mortality and negative ones suggesting negatively correlated with mortality. r7bath = Problems with bathing; r7bathcat = Dummy: Problems with bathing; r7diabs = History of diabetes mellitus; r7mobila = Impaired mobility; r7sleepr = Sleep was restless; r7sleeprcat = Dummy: Sleep was restless. PC = principal component.

**Table 3:** The leading 4-item equal-weight syndromes for mortality prediction in terms of p values.

	Variable 1	Variable 2	Variable 3	Variable 4	Mean values of the mined syndromes	Standard deviations	Regression confidents	(95% CIs)	Log(p)
**Syndrome positively associated with mortality, deadly**									
1	r7vgactx	r7ltactx	r7mobila	r7bathcat	1.66	(0.89)	0.68	(0.64 to 0.71)	Log(0)
2	r7mdactx	r7ltactx	r7mobila	r7cogimpair	1.36	(0.91)	0.67	(0.64 to 0.71)	Log(0)
3	r7mdactx	r7ltactx	r7mobila	r7bathcat	1.39	(0.97)	0.61	(0.58 to 0.64)	Log(0)
4	r7mdactx	r7lung	r7mobila	r7cogimpair	0.94	(0.74)	0.81	(0.77 to 0.85)	Log(0)
5	r7mdactx	r7lung	r7mobila	r7bathcat	0.97	(0.81)	0.71	(0.68 to 0.75)	Log(0)
6	r7mdactx	r7bath	r7mobila	r7cogimpair	0.94	(0.79)	0.76	(0.73 to 0.80)	Log(0)
7	r7mdactx	r7lift	r7mobila	r7bathcat	1.09	(0.95)	0.62	(0.59 to 0.65)	Log(0)
8	r7mdactx	r7mobila	r7memopr	r7bathcat	1.00	(0.89)	0.65	(0.62 to 0.68)	Log(0)
9	r7mdactx	r7mobila	r7underw	r7cogimpair	0.92	(0.75)	0.80	(0.76 to 0.84)	Log(0)
10	r7mdactx	r7mobila	r7underw	r7bathcat	0.95	(0.81)	0.72	(0.68 to 0.75)	Log(0)
11	r7mdactx	r7mobila	r7cogimpair	r7bathcat	1.01	(0.92)	0.64	(0.61 to 0.68)	Log(0)
12	r7mdactx	r7mobila	r7cogimpair	r7lungcat	1.02	(0.82)	0.71	(0.68 to 0.75)	Log(0)
13	r7mdactx	r7mobila	r7bathcat	r7lungcat	1.04	(0.89)	0.64	(0.60 to 0.67)	Log(0)
14	r7ltactx	r7cancr	r7mobila	r7cogimpair	0.89	(0.69)	0.84	(0.80 to 0.89)	Log(0)
15	r7ltactx	r7cancr	r7mobila	r7bathcat	0.92	(0.76)	0.74	(0.70 to 0.78)	Log(0)
16	r7ltactx	r7lung	r7bath	r7mobila	0.83	(0.63)	0.92	(0.87 to 0.96)	Log(0)
17	r7ltactx	r7lung	r7mobila	r7cogimpair	0.88	(0.70)	0.84	(0.79 to 0.88)	Log(0)
18	r7ltactx	r7lung	r7mobila	r7bathcat	0.90	(0.77)	0.73	(0.69 to 0.76)	Log(0)
19	r7ltactx	r7heart	r7mobila	r7cogimpair	0.95	(0.73)	0.81	(0.77 to 0.85)	Log(0)
**Syndromes negatively associated with mortality, death-averse**									
1	r7height	r7cogtot	r7tired	r7seizure	1.14	(0.22)	−2.03	(−2.21 to −1.86)	−111.34
2	r7bmi	r7cogtot	r7tired	r7seizure	0.95	(0.24)	−1.79	(−1.95 to −1.63)	−108.66
3	r7weight	r7cogtot	r7tired	r7seizure	0.98	(0.25)	−1.76	(−1.92 to −1.61)	−105.87
4	r7bmi	r7height	r7cogtot	r7seizure	1.50	(0.27)	−1.54	(−1.69 to −1.40)	−94.12
5	r7bmi	r7height	r7cogtot	r7tired	1.50	(0.27)	−1.54	(−1.68 to −1.39)	−93.57
6	r7height	r7weight	r7cogtot	r7seizure	1.54	(0.31)	−1.26	(−1.40 to −1.13)	−76.02
7	r7height	r7weight	r7cogtot	r7tired	1.54	(0.31)	−1.26	(−1.39 to −1.13)	−75.62
8	r7cogtot	r7tired	r7seizure	r7diabscat	0.61	(0.23)	−1.67	(−1.86 to −1.48)	−66.12
9	r7diabs	r7cogtot	r7tired	r7seizure	0.61	(0.23)	−1.67	(−1.86 to −1.48)	−66.12
10	r7bmi	r7cogtot	r7gripr	r7seizure	1.37	(0.33)	−0.94	(−1.05 to −0.83)	−65.77
11	r7cogtot	r7tired	r7gripr	r7seizure	1.01	(0.28)	−1.08	(−1.20 to −0.96)	−65.74
12	r7bmi	r7cogtot	r7tired	r7gripr	1.37	(0.33)	−0.94	(−1.04 to −0.83)	−65.56
13	r7bmi	r7cogtot	r7seizure	r7grip	1.39	(0.34)	−0.90	(−1.00 to −0.80)	−65.39
14	r7bmi	r7cogtot	r7tired	r7grip	1.39	(0.34)	−0.90	(−1.00 to −0.79)	−65.24
15	r7cogtot	r7tired	r7seizure	r7grip	1.02	(0.29)	−1.00	(−1.12 to −0.89)	−63.63
16	r7bmi	r7cogtot	r7muscle	r7seizure	0.99	(0.30)	−1.16	(−1.29 to −1.02)	−62.22
17	r7bmi	r7diabs	r7cogtot	r7seizure	0.98	(0.29)	−1.22	(−1.36 to −1.07)	−62.10
18	r7bmi	r7cogtot	r7seizure	r7diabscat	0.98	(0.29)	−1.22	(−1.36 to −1.07)	−62.10
19	r7bmi	r7weight	r7cogtot	r7seizure	1.35	(0.35)	−0.93	(−1.03 to −0.82)	−62.00
20	r7bmi	r7cogtot	r7tired	r7muscle	0.99	(0.30)	−1.16	(−1.29 to −1.02)	−61.98

CI = confidence intervals. r7bath = Problems with bathing; r7bmi = body mass index (kg/m^2^); r7bathcat = Dummy: Problems with bathing; r7cogimpair = Impaired cognition based on performance-based scores or proxy assessment; r7cogtot = Total cognition summary score; r7diabs = History of diabetes mellitus; r7diabscat = Dummy: history of diabetes mellitus; r7grip = Grip strength, largest value; r7gripl = Grip strength, left hand; r7gripr = Grip strength, right hand; r7heart = Heart problem, this wave; r7height = Height in meters; r7lift = Physical functioning: difficulty lifting 10 pounds; r7ltactx = Frequencies of light physical activities; r7lung = Lung disease, this wave; r7lungcat = Dummy: Lung disease, this wave; r7mdactx = Frequencies of moderate physical activities; r7mobila = Impaired mobility; r7muscle = Musculoskeletal problems; r7seizure = Seizures, generalized; r7sleepr = Sleep was restless; r7sleeprcat = Dummy: Sleep was restless; r7tired = tiredness all the time; r7underw = Underweight in wave 2004; r7vgactx = Frequencies of vigorous physical activities; r7weight = Weight in kilograms.

### Deadliest and most death-averse 4-item syndromes

Using the magnitudes of the regression coefficients to define the deadliest and the most death-averse 4-item equal-weight syndromes, the leading syndromes associated with mortality are listed in Table 4. The deadliest syndromes were not necessarily those with the lowest p values in Table 3.

**Table 4:** The leading 4-item equal-weight syndromes for mortality prediction in terms of regression coefficients.

Ranking	Symptom 1	Symptom 2	Symptom 3	Symptom 4	Mean	SD	Regression coefficients	(95% CIs)	Log(p)
**Syndromes negatively associated with mortality, death-averse**									
1	r7height	r7cogtot	r7tired	r7seizure	1.14	−(0.22)	−2.03	−(2.21 to −1.86)	−111.34
2	r7bmi	r7cogtot	r7tired	r7seizure	0.95	−(0.24)	−1.79	−(1.95 to −1.63)	−108.66
3	r7weight	r7cogtot	r7tired	r7seizure	0.98	−(0.25)	−1.76	−(1.92 to −1.61)	−105.87
4	r7cogtot	r7fall	r7tired	r7seizure	0.62	−(0.18)	−1.68	−(1.88 to −1.48)	−60.6
5	r7cogtot	r7tired	r7seizure	r7diabscat	0.61	−(0.23)	−1.67	−(1.86 to −1.48)	−66.12
6	r7diabs	r7cogtot	r7tired	r7seizure	0.61	−(0.23)	−1.67	−(1.86 to −1.48)	−66.12
7	r7strok	r7cogtot	r7tired	r7seizure	0.62	−(0.18)	−1.56	−(1.75 to −1.36)	−54.96
8	r7bmi	r7height	r7cogtot	r7seizure	1.5	−(0.27)	−1.54	−(1.69 to −1.40)	−94.12
9	r7bmi	r7height	r7cogtot	r7tired	1.5	−(0.27)	−1.54	−(1.68 to −1.39)	−93.57
10	r7cogtot	r7tired	r7muscle	r7seizure	0.62	−(0.25)	−1.49	−(1.67 to −1.32)	−61.62
11	r7psychs	r7cogtot	r7tired	r7seizure	0.61	−(0.21)	−1.34	−(1.53 to −1.15)	−43.27
12	r7cogtot	r7tired	r7seizure	r7psychscat	0.61	−(0.21)	−1.34	−(1.53 to −1.15)	−43.27
13	r7height	r7diabs	r7cogtot	r7seizure	1.17	−(0.27)	−1.31	−(1.47 to −1.15)	−56.12
14	r7height	r7cogtot	r7seizure	r7diabscat	1.17	−(0.27)	−1.31	−(1.47 to −1.15)	−56.12
15	r7height	r7diabs	r7cogtot	r7tired	1.17	−(0.27)	−1.3	−(1.46 to −1.14)	−55.82
16	r7height	r7cogtot	r7tired	r7diabscat	1.17	−(0.27)	−1.3	−(1.46 to −1.14)	−55.82
17	r7stroks	r7cogtot	r7tired	r7seizure	0.61	−(0.22)	−1.3	−(1.48 to −1.11)	−42.14
18	r7height	r7weight	r7cogtot	r7seizure	1.54	−(0.31)	−1.26	−(1.40 to −1.13)	−76.02
19	r7height	r7weight	r7cogtot	r7tired	1.54	−(0.31)	−1.26	−(1.39 to −1.13)	−75.62
20	r7height	r7cogtot	r7fall	r7seizure	1.18	−(0.22)	−1.25	−(1.42 to −1.08)	−44.75
**Syndrome positively associated with mortality, deadly**									
1	r7height	r7bath	r7tired	r7seizure	0.6	−(0.16)	2.1	(1.92 to 2.29)	−109.75
2	r7bath	r7fall	r7tired	r7seizure	0.08	−(0.17)	2.09	(1.94 to 2.23)	−166.48
3	r7strok	r7bath	r7tired	r7seizure	0.07	−(0.17)	1.98	(1.84 to 2.13)	−154.7
4	r7height	r7bath	r7fall	r7seizure	0.63	−(0.20)	1.84	(1.70 to 1.98)	−140.51
5	r7height	r7bath	r7fall	r7tired	0.63	−(0.20)	1.83	(1.69 to 1.97)	−139.52
6	r7bath	r7tired	r7seizure	r7actsum	0.72	−(0.32)	1.82	(1.71 to 1.92)	−239.62
7	r7fall	r7tired	r7seizure	r7actsum	0.72	−(0.28)	1.82	(1.70 to 1.94)	−182.66
8	r7toilt	r7fall	r7tired	r7seizure	0.07	−(0.16)	1.79	(1.64 to 1.95)	−113.35
9	r7dress	r7fall	r7tired	r7seizure	0.08	−(0.17)	1.78	(1.64 to 1.92)	−132.36
10	r7height	r7strok	r7bath	r7seizure	0.62	−(0.20)	1.78	(1.64 to 1.92)	−133.53
11	r7height	r7strok	r7bath	r7tired	0.62	−(0.20)	1.77	(1.63 to 1.91)	−132.64
12	r7toilt	r7tired	r7seizure	r7actsum	0.71	−(0.30)	1.76	(1.65 to 1.87)	−208.02
13	r7height	r7dress	r7tired	r7seizure	0.6	−(0.17)	1.75	(1.57 to 1.92)	−84.4
14	r7height	r7tired	r7seizure	r7actsum	1.23	−(0.26)	1.73	(1.59 to 1.86)	−137.23
15	r7strok	r7tired	r7seizure	r7actsum	0.71	−(0.29)	1.71	(1.59 to 1.83)	−175.87
16	r7strok	r7bath	r7fall	r7tired	0.11	−(0.21)	1.7	(1.58 to 1.81)	−171.02
17	r7strok	r7bath	r7fall	r7seizure	0.11	−(0.21)	1.7	(1.58 to 1.82)	−171.71
18	r7strok	r7dress	r7tired	r7seizure	0.08	−(0.18)	1.7	(1.56 to 1.84)	−123.24
19	r7dress	r7tired	r7seizure	r7actsum	0.73	−(0.32)	1.69	(1.58 to 1.79)	−219.62
20	r7height	r7bath	r7seizure	r7actsum	1.27	−(0.32)	1.69	(1.58 to 1.79)	−223.7

CI = confidence interval; SD = standard deviation. r7actsum = Summary scores of physical activities; r7bath = Problems with bathing; r7bmi = body mass index (kg/m^2^); r7bathcat = Dummy: Problems with bathing; r7cogimpair = Impaired cognition based on performance-based scores or proxy assessment; r7cogtot = Total cognition summary score; r7diabs = History of diabetes mellitus; r7diabscat = Dummy: history of diabetes mellitus; r7dress = Problem getting dressed; r7fall = Falls; r7grip = Grip strength, largest value; r7gripl = Grip strength, left hand; r7gripr = Grip strength, right hand; r7heart = Heart problem, this wave; r7height = Height in meters; r7lift = Physical functioning: difficulty lifting 10 pounds; r7ltactx = Frequencies of light physical activities; r7lung = Lung disease, this wave; r7lungcat = Dummy: Lung disease, this wave; r7mdactx = Frequencies of moderate physical activities; r7mobila = Impaired mobility; r7muscle = Musculoskeletal problems; r7psychs = Psychiatric problem since last wave; r7seizure = Seizures, generalized; r7sleepr = Sleep was restless; r7 sleeprcat = Dummy: Sleep was restless; r7strok = Stroke, this wave; r7toilt = Toileting problem; r7tired = tiredness all the time; r7underw = Underweight in wave 2004; r7vgactx = Frequencies of vigorous physical activities; r7weight = Weight in kilograms.

### The role of input symptoms in mined syndromes

Figure 3 shows the frequencies of input variables constituting significant syndromes regarding the prediction of mortality. The input variables were listed on the horizontal axis according to the serial numbers given during data processing (Appendix 2). Due to the equal weighting and the equal chances of constituting a syndrome, the frequencies of constituting all 4-item syndromes were the same for all input symptoms, 54,740 or 0.057% of all syndromes, as expected. The frequencies of constituting PC-based syndromes were not consistent across the input symptoms. However, it was not expected that the frequencies of symptoms constituting significant 4-item equal-weight syndromes were also similar to the pattern of eligible input symptoms in Fig. 3. The frequencies of constituting significant PC-based syndromes were proportional to those of constituting all syndromes.

The five most frequent input symptoms constituting all or significant PC-based or 4-item equal-weight syndromes are listed in Table 5. The variables were ordered according to the frequencies of constituting all significant syndromes and deadly or death-averse syndromes. The leading variables constituting PC-based syndromes were “tiredness all the time”, “musculoskeletal problems”, “grip strength”, “malignant disease”, and “history of malignant disease”. Three variables that constituted the death-averse PC-based syndromes the most frequently were “tiredness all the time”, “malignant disease” and “musculoskeletal problems”.

**Table 5:** The proportions of individual variables consisting all or significant syndromes: principal component-based or equal-weight 4-item syndromes.

Most frequent variables in significant syndromes (order, frequencies, proportions relative to significant syndromes, proportions relative to all syndromes)	PC-based syndromes	Equal-weight 4-item syndromes
1	Tiredness all the time (variable name = r7tired)	(i) Felt that everything I did was an effort in last week. (variable name = r7effort)
Frequencies of constituting significant syndromes	1799	54740
Proportions relative to significant syndromes	0.643	0.057
Proportions relative to all syndromes	0.357	0.056
2	Musculoskeletal problems (variable name = r7muscle)	(ii) Could not get going in last week. (variable name = r7going)
Frequencies of constituting significant syndromes	1619	54740
Proportions relative to significant syndromes	0.578	0.057
Proportions relative to all syndromes	0.321	0.056
3	Grip strength, right hand (variable name = r7gripr)	Frequencies of moderate physical activities (variable name = r7mdactx)
Frequencies of constituting significant syndromes	1555	54740
Proportions relative to significant syndromes	0.556	0.057
Proportions relative to all syndromes	0.308	0.056
4	Dummy: Malignant disease (variable name = r7cancrcat)	Frequencies of light physical activities (variable name = r7ltactx)
Frequencies of constituting significant syndromes	1553	54740
Proportions relative to significant syndromes	0.555	0.057
Proportions relative to all syndromes	0.308	0.056
5	Malignant disease (variable name = r7cancr)	Impaired mobility (variable name = r7mobila)
Frequencies of constituting significant syndromes	1519	54740
Proportions relative to significant syndromes	0.543	0.057
Proportions relative to all syndromes	0.301	0.056
**Deadly syndromes only**		
Most frequent variables in deadly syndromes (order, proportions relative to significant syndromes, proportions relative to all syndromes)	PC-based syndromes	Equal-weight 4-item syndromes
1	Tiredness all the time (variable name = r7tired)	(i) Felt that everything I did was an effort in last week. (variable name = r7effort)
Frequencies of constituting significant syndromes	804	54740
Proportions relative to significant syndromes	0.287	0.057
Proportions relative to all syndromes	0.159	0.056
2	Musculoskeletal problems (variable name = r7muscle)	(ii) Could not get going in last week. (variable name = r7going)
Frequencies of constituting significant syndromes	749	54740
Proportions relative to significant syndromes	0.268	0.057
Proportions relative to all syndromes	0.149	0.056
3	Grip strength, right hand (variable name = r7gripr)	Frequencies of moderate physical activities (stratified according to sex) (variable name = r7mdactx)
Frequencies of constituting significant syndromes	704	54740
Proportions relative to significant syndromes	0.252	0.057
Proportions relative to all syndromes	0.14	0.056
4	Malignant disease (variable name = r7cancr)	Frequencies of light physical activities (stratified according to sex) (variable name = r7ltactx)
Frequencies of constituting significant syndromes	697	54740
Proportions relative to significant syndromes	0.249	0.057
Proportions relative to all syndromes	0.138	0.056
5	Dummy: malignant disease (variable name = r7cancrcat)	Impaired mobility (variable name = r7mobila)
Frequencies of constituting significant syndromes	681	54740
Proportions relative to significant syndromes	0.243	0.057
Proportions relative to all syndromes	0.135	0.056
**Death-averse syndromes only**		
Most frequent variables in death-averse syndromes (order, proportions relative to significant syndromes, proportions relative to all syndromes)	PC-based syndromes	Equal-weight 4-item syndromes
1	Tiredness all the time (variable name = r7tired)	History relevant to cognitive impairment or loss (variable name = r7cogtot)
Frequencies of constituting significant syndromes	995	4028
Proportions relative to significant syndromes	0.355	0.004
Proportions relative to all syndromes	0.197	0.004
2	Dummy: Malignant disease (variable name = r7cancrcat)	Body mass index: kg/m^2^ (variable name = r7bmi)
Frequencies of constituting significant syndromes	872	765
Proportions relative to significant syndromes	0.312	0.001
Proportions relative to all syndromes	0.173	0.001
3	Musculoskeletal problems (variable name = r7muscle)	Grip strength, largest value(variable name = r7grip)
Frequencies of constituting significant syndromes	870	740
Proportions relative to significant syndromes	0.311	0.001
Proportions relative to all syndromes	0.173	0.001
4	Weight in wave 2002 minus weight in wave 2004 !10% of weight in wave 2002 or body mass index o18.5 kg/m2 (variable name = r7frail1_2)	Weight in kilograms (variable name = r7weight)
Frequencies of constituting significant syndromes	867	735
Proportions relative to significant syndromes	0.31	0.001
Proportions relative to all syndromes	0.172	0.001
5	Time to walk 8 ft, converted to time to walk 15 ft. Cutoff criteria according to sex and height remain the same (variable name = r7frail3_4)	Grip strength, right hand (variable name = r7gripr)
Frequencies of constituting significant syndromes	867	688
Proportions relative to significant syndromes	0.31	0.001
Proportions relative to all syndromes	0.172	0.001

The input symptoms that constituted significant 4-item syndromes were “felt everything I did was an effort in last week”, “could not get going in last week”, “low energy expenditure”, and “impaired mobility”. These input symptoms were also the leading variables constituting significantly deadly 4-item syndromes. However, the leading symptoms constituting significantly death-averse 4-item syndromes were “history relevant to cognitive impairment or loss”, “weight loss”, “weakness measured by grip strengths: the weakest 20%”, “weight loss measured by weight in wave 2002 subtracted from weight in wave 2004” and “grip strength, right hand”.

### Relationship between input symptoms and mined syndromes

The associations between input symptom characteristics and the statistical significance of mined syndromes were explored, presented in Appendix 3. The characteristics of the input symptoms studied were statistically significant for mortality prediction (p values), variances, and regression coefficients for mortality prediction. The frequencies of achieving statistically significant mined syndromes were classified by weighting schemes (equal-weighted or PC-based) and directions of mortality prediction (all, deadly, and death-averse). There were 18 (3 × 2 × 3) associations studied. Among the associations, ten were significant. Input symptom p values in log scale were significantly associated with the frequencies of constituting significant PC-based syndromes (adjusted R-squared = 0.15, p < 0.0001), significantly deadly PC-based syndromes (adjusted R-squared = 0.25, p < 0.0001), and significantly death-averse 4-item equal-weight syndromes (adjusted R-squared = 0.12, p = 0.01). The input symptom variances were associated with the frequency of constituting significant 4-item equal-weight syndromes (adjusted R-squared = 0.08, p = 0.01) and deadly 4-item equal-weight syndromes (adjusted R-squared = 0.09, p < 0.01). The regression coefficients of the input symptoms predicting mortality were associated with the frequencies of constituting significant PC-based syndromes (adjusted R-squared = 0.04, p < 0.05), significant equal-weight 4-item syndromes (adjusted R-squared = 0.53, p < 0.001), significant deadly PC-based syndromes (adjusted R-squared = 0.11, p < 0.01), significant deadly equal-weight 4-item syndromes (adjusted R-squared = 0.50, p < 0.001), and significant death-averse 4-item equal-weight syndromes (adjusted R-squared = 0.48, p < 0.001).

## Discussion

Traditionally, frailty syndromes have been proposed incrementally and supported mostly by significant statistical correlation with major outcomes^11,14,24,25^. The results of this study provide important insight into the generation of significant frailty syndromes and how to examine newly created frailty syndromes.

First, it is relatively easy to generate frailty syndromes significantly associated with mortality with previously defined frailty symptoms using the HRS data. The chance of discovering significant equal-weight 4-item and PC-based frailty syndromes is 99.3% and 55.5%, respectively. By only focusing on deadly frailty syndromes (positive coefficients regarding mortality prediction), the chances of discovering significant 4-item and PC-based frailty syndromes are 98.8% and 25.2%, respectively. This finding is supported by a previous publication, an index-mining article that identified more than 6,000 frailty syndromes with lower p values for mortality prediction than three commonly used frailty syndromes^12^.

Conventionally, researchers have claimed to identify individual new frailty syndromes based on the approach we have demonstrated. Numerous frailty syndromes are defined and created by summing or aggregating various frailty symptoms^13,24,26^. However, if scrutinized, frailty syndromes may fail to represent the theories on which they are based^12^. Frailty symptoms cannot fully explain frailty syndromes due to data manipulation and inclusion of highly correlated symptoms^12^. Based on the results in this study, it is fairly easy to find many frailty syndromes statistically significantly correlated with major outcomes^27^. With a number of symptoms that become more prevalent among the elderly and equal weighting, the success rates of finding a 4-item frailty syndrome significantly associated with mortality is more than 98%. Our data-driven approach highlights the problem of relying on statistical significance for screening and selection of newly developed indices or syndromes.

Second, it is possible to find the deadliest and the most death-averse syndromes based on p values and regression coefficients for equal-weight 4-item syndromes. We have found it less convincing to compare the PC-based syndromes based on the coefficients regarding mortality prediction, although many of them have p values approximately zero. The PC-based syndromes are very likely to have mean values approaching zero and are difficult to interpret.

Third, the input symptoms are not equally important in forming PC-based or equal-weight syndromes that significantly predict mortality. For PC-based syndromes, some input variables constitute significant syndromes more often than others, especially those assigned with the loadings of the first PC. Fourth, three characteristics of the input symptoms are important for creating frailty syndromes that are significantly associated with mortality: p values for mortality prediction in log scales, symptom variances, and coefficients for mortality prediction (Appendix 3). Based on statistical significance, the regression coefficients of input symptoms for mortality prediction the best predict the frequencies of constituting significant PC-based or 4-item syndromes. However, the exact reasons for the importance of these characteristics are not clear. The relationships between input symptoms and the significance of mined syndromes require further research.

### What are the deadliest and the most death-averse frailty syndromes?

For PC-based syndromes, the loadings of the first PC are associated highly with significant deadly syndromes, i.e. those with positive regression coefficients for mortality prediction. This seems reasonable because the first PC accounted for the most variances of all variables^28,29^. In contrast, out of total 71 PCs, the loadings of 68^th^ PC can lead to significant death-averse syndromes, i.e. those with negative regression coefficients for mortality prediction. Overall, the PCA loadings can lead to frailty syndromes with very small mean values and large regression coefficients, especially for the leading PC-based syndromes. It can make the interpretation of the regression coefficients very difficult. Therefore, we did not search for the deadliest or the most death-averse PC-based syndromes according to regression coefficients.

By applying equal weights to input symptoms, there can be a large number of equal-weight syndromes to be mined. For the 4-item equal-weight syndromes, the mean values and regression coefficients are within ranges that are easier to understand. For a given equal-weight syndrome, we can estimate the magnitude of mortality risk increase due to the occurrence of one or more input symptoms. With interpretable values and coefficients, we considered the equal-weight syndromes ideal to search for the deadliest and the most death-averse frailty syndromes based on regression coefficients. The most death-averse 4-item syndrome in terms of regression coefficients consists of height, history relevant to cognitive impairment or loss, tiredness all the time, and generalized seizure. The deadliest 4-item syndrome consists of height, problem with bathing, tiredness all the time, and generalized seizure.

### Association between input symptoms and significant syndromes

To our knowledge, there is no research on syndrome mining and what characteristics of input symptoms are associated with significant syndromes. We first plotted the frequencies of constituting all and significant syndromes by input symptoms for PC-based and 4-item syndromes. At the first glance, it was found that the frequencies of constituting PC-based syndromes varied across input symptoms because PCA rotated the matrix in order to maximize the variances of the first PCs^28,29^. Some variables have been given more weight to constitute the first PC that explained the most variances in the data set^28,29^. The rich information in terms of variances in the first PC may be the reason why most of the syndromes weighted by the loadings of the first PC have the smallest p values for the prediction of mortality. In contrast, the loadings of the 68^th^ PC were also associated with the several of the leading PC-based syndromes. The exact reason to this is not clear and we think the PCs other than the first one may need to be examined empirically.

In addition to PC loadings, the significance of the input symptoms may also play a role in significant PC-based syndromes. Among three characteristics of the input symptoms, significance to predict mortality, symptom variances, and coefficients to predict mortality, we notice that the frequencies of constituting significant PC-based syndromes can be best explained by the p values of the input symptoms. However, the disadvantage of PC-based syndromes is the difficulty of selecting the deadliest and the most death-averse syndromes based on the regression coefficients.

For the 4-item syndromes, we have very different conclusions. In general, the frequencies of constituting significant equal-weight syndromes are more evenly distributed across input symptoms than PC-based syndromes. This suggests chance alone is an important factor to find significant 4-item equal-weight syndromes. If studying further, we found there are other things at play, especially the regression coefficients of the input symptoms. By plotting the frequencies of constituting significant syndromes and input symptom coefficients, we can find that there are input symptoms with coefficients larger than two or smaller than −2 and the frequencies can be best explained by the coefficients. The coefficients of the input symptoms can not only well explain the frequencies of constituting significant 4-item equal-weight syndromes, but also those of the deadliest and the most death-averse. If focusing on the deadliest and the most death-averse, we found that the leading syndromes were often the syndromes consisting of an input symptom with a regression coefficient larger than two or smaller than −2, along with three other input symptoms with coefficients close to zero. If this is applicable to other data sets, researchers will be able to produce as many deadly or death-averse syndromes as possible by summing one input symptom with large regression coefficients for mortality prediction and other symptoms that have coefficients close to zero.

### Recommendations regarding innovative and existing frailty syndromes

For readers interested in frailty syndromes, the findings have important implications. To address them, recommendations are made for new researchers to understand the value of innovative and existing frailty syndromes. First, before frailty can be diagnosed with pathological evidence or diagnostic tests, readers should expect more frailty syndromes being “discovered” and presenting statistically significant correlations with major outcomes. The ease of finding one and the incentive to publish can be important factors. We have demonstrated the success rate of identifying a significant deadly 4-item frailty syndrome could be higher than 98%, when single syndromes were proposed and tested individually. Researchers can be incentivized to generate new syndromes because publications are important for researchers to secure tenure and research grants^20,30^. Readers should proceed with caution in using any frailty syndromes.

Second, readers are strongly advised to appraise existing innovative frailty syndromes critically. Three of the most widely accepted frailty syndromes are biased and fail to represent their own theoretic frameworks^12^. We propose a review framework for indices and composite measures, particularly frailty syndromes^12^. The key aspects to understanding the value of novel indices include interpretability, input symptom selection, assumptions, and the disclosure of data processing and transformation^12^. When innovative syndromes are used to predict major outcomes, the predictive power of the syndromes must be compared with that of the input symptoms^12^.

Third, we recommend searching for single interpretable measures before adopting composite measures or syndromes that are diagnosed based on multiple rules or criteria. Without raw data, it is often difficult to assess biases or assumptions in the process of data transformation^12^. One example is the subjective scale we proposed for the detection of frailty^18^. We proposed this frailty scale because we aimed to avoid the problematic assumptions and the questionable interpretability issue in three commonly used frailty syndromes^12^. These issues damage the validity of these frailty syndromes and a new scale is necessary for future frailty research.

Fourth, we recommend comparing the predictive power of the input variables or symptoms with the syndromes regarding any outcomes. This is because the predictive power of the input variables is likely to be better than that of the syndromes, indicated in the results^12^. In addition, there are at least 6,000 alternative 4-item syndromes that better predict mortality better than the three most commonly used frailty syndromes^12^. Lastly, we think readers are key to disseminate and promote research results. We suggest that the public use social media and other means to discuss and debate innovative research findings, especially those based on composite diagnostic criteria^31,32^.

### Strengths and limitations

This study’s strengths rest in the use of a large and public data set for analysis, peer-reviewed methods^12^, the application of index mining techniques^20^, long-term follow-up of individuals^12,33^, and the use of input variables well recognized in aging research^12^. However, there are several limitations. First, we tried only two methods to mine innovative frailty syndromes, principal component analysis (PCA) and equal weighting, despite the large number of frailty syndromes mined. Other methods to generate composite measures^20^ may need to be tested in the future. Second, the criteria to determine the importance of the mined syndromes can be refined. In this study, we use p values and regression coefficients. We think there are other opportunities to produce syndromes that better address clinicians’ need^18^. Third, more research on syndrome mining or index mining is required. Currently, there is a framework to guide the creation of composite measures or indices and an appraisal tool proposed for the critical appraisal and reporting of innovative indices^20,23^. However, there is still a need for more guidance. Lastly, the computation and screening of PC-based syndromes are much more efficient than equal-weight ones. This is because the number of all possible combinations of equal-weight syndromes is astronomical, and other equal-weight syndromes can be tested. Researchers are advised to prepare for the computational demand to examine other equal-weight syndromes.

## Conclusion

The application of index mining in medicine helps to identify new frailty syndromes based on the 72 symptoms previously used to define frailty among the HRS participants. Two approaches have been adopted, PC-based and equal-weight 4-item syndromes to generate frailty syndrome for mortality prediction. We notice that both approaches can help to discover frailty syndromes negatively or positively associated with mortality, death-averse or deadly syndromes respectively. There are far more equal-weight 4-item syndromes, and the syndromes with the least p values are positively associated with mortality, i.e. deadly. Syndromes are ranked according to p values and regression coefficients for mortality prediction. Because some of the PCA loadings can be close to zero, there are PC-based syndromes with means close to zero and extremely large regression coefficients.

For this reason, only equal-weight 4-item syndromes have been ranked based on regression coefficients. The significance of the input symptoms to predict mortality seems to be associated with the frequencies of constituting significant syndromes. Chance plays an important role in forming significant 4-item equal-weight syndromes because all input symptoms consist of more than 50,000 significant syndromes. The variations in the frequencies of constituting significant, deadly or death-averse equal-weight 4-item syndromes can be partly explained by the regression coefficients or the statistical significance of the input symptoms regarding mortality prediction. To form the deadliest and the most death-averse 4-item frailty syndromes in terms of regression coefficients, it often takes an input symptom with a regression coefficient larger than two or smaller than −2, along with three other input symptoms with coefficients close to zero. Based on the findings, with proper design, planning and execution, a large number of innovative frailty syndromes significantly associated with major outcomes can be searched and identified. We suggest readers critically appraise innovative syndromes by assessing syndrome interpretability, assumptions, disclosure of data processing, and differences in predictive power between novel syndromes and their input symptoms.

## Methods

We created frailty syndromes with 72 frailty symptoms in the HRS and searched for the syndromes associated with mortality with positive or negative coefficients. The characteristics of input symptoms were analyzed for the associations with the p values and the regression coefficients of the newly created frailty syndromes. The syndrome scores were calculated based on two methods. One method was to weight the input symptoms by the PCA loadings^20^. The other was to assign equal weights to generate syndromes^23^. With computational constraints discussed below, 4-item syndromes were generated.

### Symptom search

In brief, the HRS was first implemented in 1996 and followed adults aged 50 years and over every two years in the United States^34^. The longitudinal HRS data set with contribution from RAND Corporation, version P, was used^33,34^. This longitudinal data set was created by merging and integrating all 2-year waves since 1996 and most of the variables in the original waves were retained^35^. Some of the frailty symptoms that existed only in the 2004 wave were retrieved from original 2-year cross-sectional data^11^. The list of frailty syndromes can be retrieved online (10.1371/journal.pone.0197859.s002) and in Appendix 2^12^. The design and the history of the HRS could be found elsewhere^34^.

Frailty symptoms were the input or domain variables of three frailty indices: the Functional Domain model proposed by Strawbridge *et al*.^24^, the Burden model by Rockwood *et al*.^15^, and the Biological Syndrome model by Fried *et al*.^11,26^. Domain variables were the intermediate variables required to generate frailty indices^23^. These variables met the requirements recommended by the authors of the Burden Model in Searle *et al*.^14^.

To produce the three frailty syndromes, four, one and five domain variables (intermediate variables derived from input symptoms) were required respectively^12^. The domain variables were created based on nine, 25 and ten input variables respectively^12^. For example, four functional domains were required to be summed to create the frailty index proposed by Strawbridge *et al*.^24^.

### Principal component analysis (PCA)

PCA had been widely used for dimension reduction or data pre-processing^36^. There are several variants of PCA, such as linear, supervised and kernel PCA^36–38^, and similar data processing techniques, such as independent component analysis^28,39^. We found that there were limited choices of dimension reduction methods applicable to survey design^40^. Linear PCA was considered an optimal option for our research involving survey data^41^. The PCA loadings were retrieved after PCA. The details in the PCA could be found elsewhere^20^.

### Principal component-based syndromes

In input symptoms or conditions, a value of one represented the existence of the symptoms and zero indicated the absence of the symptoms^12^. Syndrome scores were the numerical sums of input symptoms weighted equally or by the PCA loadings. The process was similar to the indices or composite measures identified through index mining, a science with an aim to improve methods to design, generate, and validate composite measures and indices^20,23,42,43^. The PCA loadings weighted input variables to generate PCs linearly^20,44^. The number of PCs was the same as the number of input variables, denoted by *N*. In Eq. 1, a PC, specified with a subscript *pc*, was the sum of all input variables, denoted by *x*, weighted by PC-specific loadings, denoted by *L*.

1 $$P{C}_{pcn}=\mathop{\sum }\limits_{i=1}^{N}{L}_{i}{x}_{i}$$

The process of PCA-based syndrome score generation is as follows. The first syndrome score for each PC, denoted by *Syndrome*_*pc.n*_, is the product of the leading variable, in terms of absolute loadings. *L*_*i*_*x*_*i*_ denotes PC-specific loading, while *pc.n* refers to the PC that was used to produce syndrome scores and *n* (n equals one) specifying the numbers of input variables required for the index in Eq. 2. The second syndrome score is the sum of the products of the first two leading symptoms weighted by PC-specific loadings, denoted by ${\sum }_{i=1}^{2}{L}_{i}{x}_{i}$. By repeating the same procedure, we included all variables weighted by loadings in each PC and the last syndrome score in each PC was the same as the PC value. One of the 72 input variables was excluded for zero variance (r7seizure, generalized seizure). There were 71 weighted indices generated for each PC, 5,041 for 71 PCs in total.

2 $$Syndrom{e}_{pc.n}=\mathop{\sum }\limits_{i=1}^{n}{L}_{i}{x}_{i}$$

### Equal-weight 4-item syndromes

Any four of the 72 input symptoms and conditions were summed to generate 1,028,790 equal-weight 4-item frailty syndromes^23^. One of the aforementioned frailty models, the Functional Domains Model, used only four input variables^12^. The use of indices consisting of less than four variables was rare. Only 4-item syndromes were generated and tested because the number of all possible syndromes, 2^72^-73, surpassed our computational resources^23^. The computation time for five or more-item syndromes exceeded six months on an ordinary desktop computer. As a first attempt, only four of the 72 input symptoms or conditions were summed to represent a syndrome.

### Discrete-time survival analysis

Survival analysis was used to understand the predictive power of each syndrome on mortality. The outcome variable was mortality among HRS participants interviewed in 2004. Follow-up time ranged from one to 13 years for this cohort^12^. Discrete-time survival analysis was adopted because of the violation of the proportional hazard assumption of the Cox survival model^45^. Mortality risks were estimated with or without controlling for sex, race/ethnicity, education, per capita income, and per capita wealth^12,45^. We documented the predictive power using p values of alternative indices, model Akaike information criterion (AIC) values, and residual deviances relative to null models were documented^28^. Two-tailed p values less than 0.05 were considered statistically significant. All statistical analyses and data processing were conducted with R (v3.31)^46^ and RStudio (v1.0.44)^47^. Benjamini and Hochberg method was used when p values were adjusted for multiple comparisons^48^.

### Factors associated with statistically significant frailty syndromes

The relationship between input symptoms and mined syndromes were analyzed in two ways. First, we tested the association between the frequencies of significant syndromes and the p values of input symptoms that predict mortality. Next, we analyzed the associations between the frequencies of significant syndromes and the variances of input symptoms. The strength of associations was determined with adjusted R-squared and model p values.

### Ethics approval

This secondary data analysis was approved by the ethics review committee at the Centre Hospitalier de l’Université de Montréal (project number: CE 15.115) that also waived the need for written informed consent from the HRS participants that we are unable to identify. All methods were performed in accordance with the guidelines and regulations relevant to the analysis of public data.

### Consent to participate

The HRS participants consented to the study before being interviewed. The same ethics committee at the Centre Hospitalier de l’Université de Montréal approved the protocol and waived the need for written informed consent from the HRS participants that we are unable to identify.

## Supplementary information

Supplemental materials.

## Data Availability

The HRS data produced by the RAND Center for the Study of Aging can be accessed via the University of Michigan site (https://hrs.isr.umich.edu/data-products). The authors do not have special access to the HRS data. The authors do not have access to identifying patient data and are unable to retrieve patients’ identification. The data available to the authors are anonymized and there are no identifiers available.
