# Supplementary material for: Using syndrome mining with the Health and Retirement Study to identify the deadliest and least deadly frailty syndromes
Source: Sci Rep. 2020 Apr 8;10:5357. doi: 10.1038/s41598-020-60869-8 (PMC7142157; doi:10.1038/s41598-020-60869-8)

Using syndrome mining with the Health and Retirement Study to identify the deadliest and least deadly frailty syndromes

Authors

Yi-Sheng Chao, Chao-Jung Wu, Hsing-Chien Wu, Hui-Ting Hsu, Lien-Cheng Tsao, Yen-Po Cheng, Yi-Chun Lai, Wei-Chih Chen, MD

Supplemental material 1

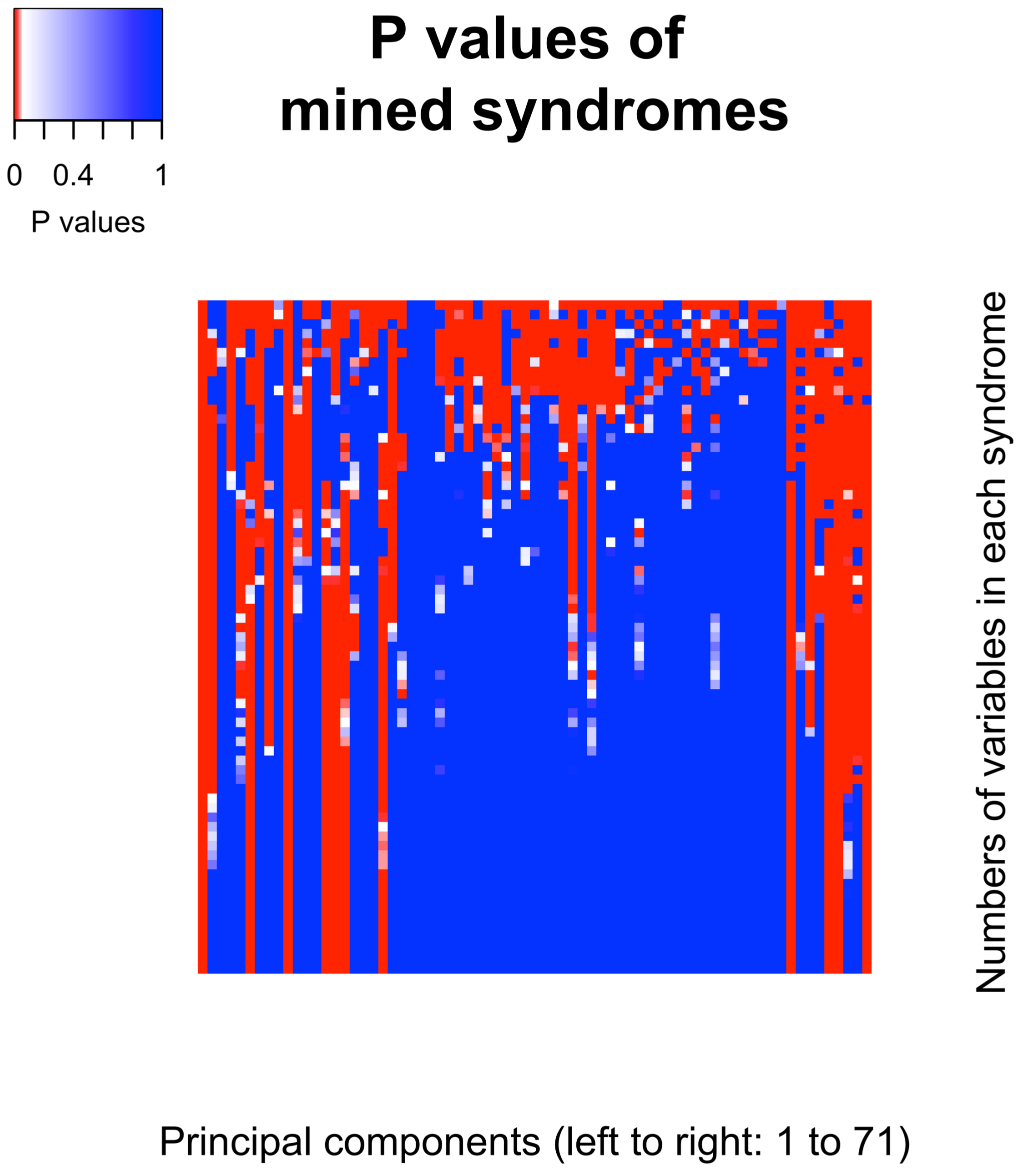
P values of
mined syndromes
0
0.4
1
P values
Numbers of variables in each syndrome
(top to down: 1 to 71)
Principal components (left to right: 1 to 71)

Using syndrome mining with the Health and Retirement Study to identify the deadliest and least deadly frailty syndromes

Authors

Yi-Sheng Chao, Chao-Jung Wu, Hsing-Chien Wu, Hui-Ting Hsu, Lien-Cheng Tsao, Yen-Po Cheng, Yi-Chun Lai, Wei-Chih Chen, MD

Supplemental material 2

Variable names	Imported from 2004 wave HRS data	Numbers of missing values among those eligible for the first frailty index	Proportions of missing values relative to those eligible for the first frailty index	Numbers of missing values among those eligible for the second frailty index	Proportions of missing values relative to those eligible for the second frailty index	Numbers of missing values among those eligible for the third frailty index	Proportions of missing values relative to those eligible for the third frailty index	Original RAND HRS variables	frail1	frailcat1	frail2	frailcat2	frail3	frailcat3	Variable definitions	All possible responses (if categorical variables)
**r7depres**		1248	0.112300908845496	954	0.123687281213536	0	0	TRUE	TRUE	TRUE	TRUE	TRUE			Feeling sad, blue, depressed	0.no; 1.yes
**r7effort**		1254	0.1128408170611	959	0.124335537404382	1	0.000609013398294762	TRUE	TRUE	TRUE	TRUE	TRUE	TRUE	TRUE	(i) Felt that everything I did was an effort in last week.	0.no; 1.yes
**r7sleepr**		1245	0.112030954737695	954	0.123687281213536	0	0	TRUE	TRUE	TRUE	TRUE	TRUE	TRUE	TRUE	Sleep changes	0.no; 1.yes
**r7going**		1264	0.113740664087105	969	0.125632049786075	1	0.000609013398294762	TRUE	TRUE	TRUE	TRUE	TRUE	TRUE	TRUE	(ii) Could not get going in last week.	0.no; 1.yes
**r7vgactx**		10	0.000899847026005579	9	0.00116686114352392	3	0.00182704019488429	TRUE	TRUE	TRUE	TRUE	TRUE	TRUE	TRUE	Low energy expenditure: Frequency of three intensities of activity, lowest quintile (stratified according to sex)	2.>1 per week; 3.1 per week; 5.never; 4.l-3 per mon; 1.every day
**r7mdactx**		9	0.000809862323405021	5	0.000648256190846623	1	0.000609013398294762	TRUE	TRUE	TRUE	TRUE	TRUE	TRUE	TRUE	Low energy expenditure: Frequency of three intensities of activity, lowest quintile (stratified according to sex)	4.l-3 per mon; 2.>1 per week; 5.never; 3.1 per week; 1.every day
**r7ltactx**		6	0.000539908215603347	3	0.000388953714507974	1	0.000609013398294762	TRUE	TRUE	TRUE	TRUE	TRUE	TRUE	TRUE	Low energy expenditure: Frequency of three intensities of activity, lowest quintile (stratified according to sex)	3.1 per week; 2.>1 per week; 5.never; 4.l-3 per mon; 1.every day
**r7bmi**		161	0.0144875371186898	113	0.0146505899131337	10	0.00609013398294762	TRUE	TRUE	TRUE	TRUE	TRUE	TRUE	TRUE	Weight loss: BMI o18.5 kg/m2	
**r7height**		18	0.00161972464681004	17	0.00220407104887852	1	0.000609013398294762	TRUE	TRUE	TRUE	TRUE	TRUE	TRUE	TRUE		
**r7weight**		143	0.0128678124718798	96	0.0124465188642552	9	0.00548112058465286	TRUE	TRUE	TRUE	TRUE	TRUE	TRUE	TRUE	Weight loss: Weight in wave 2002 minus weight in wave 2004 >10% of weight in wave 2002	
**r7hibp**		20	0.00179969405201116	18	0.00233372228704784	1	0.000609013398294762	TRUE	TRUE	TRUE	TRUE	TRUE	TRUE	TRUE	Arterial hypertension	0.no; 1.yes; 4.disp prev record and no cond; 3.disp prev record and has cond
**r7diabs**		79	0.00710879150544407	50	0.00648256190846623	0	0	TRUE	TRUE	TRUE	TRUE	TRUE	TRUE	TRUE	History of diabetes mellitus	0.no; 1.yes
**r7cancr**		10	0.000899847026005579	5	0.000648256190846623	0	0	TRUE	TRUE	TRUE	TRUE	TRUE	TRUE	TRUE	Malignant disease	0.no; 1.yes; 4.disp prev record and no cond; 3.disp prev record and has cond
**r7lung**		10	0.000899847026005579	8	0.0010372099053546	1	0.000609013398294762	TRUE	TRUE	TRUE	TRUE	TRUE	TRUE	TRUE	Lung problems or Respiratory problems	0.no; 1.yes; 4.disp prev record and no cond; 3.disp prev record and has cond
**r7heart**	TRUE	15	0.00134977053900837	14	0.00181511733437054	1	0.000609013398294762	TRUE	TRUE	TRUE	TRUE	TRUE	TRUE	TRUE	Congestive heart failure	1.yes; 0.no; 4.disp prev record and no cond; 3.disp prev record and has cond
**r7strok**		6	0.000539908215603347	4	0.000518604952677298	0	0	TRUE	TRUE	TRUE	TRUE	TRUE	TRUE	TRUE	Cerebrovascular problems	0.no; 1.yes; 4.disp prev record and

																no cond; 2.tia/possible stroke; 3.disp prev record and has cond
**r7stroke**		12	0.00107981643120669	6	0.000777907429015947	0	0	TRUE	TRUE	TRUE	TRUE	TRUE	TRUE	TRUE	History of stroke	0.no; 1.yes
**r7stroks**		69	0.0062089444794385	42	0.00544535200311163	0	0	TRUE	TRUE	TRUE	TRUE	TRUE				0.no; 1.yes
**r7psych**		13	0.00116980113380725	9	0.00116686114352392	0	0	TRUE	TRUE	TRUE	TRUE	TRUE	TRUE	TRUE	Depression (clinical impression)	0.no; 1.yes; 4.disp prev record and no cond; 3.disp prev record and has cond
**r7psychs**		73	0.00656888328984073	48	0.00622325943212758	0	0	TRUE	TRUE	TRUE	TRUE	TRUE	TRUE	TRUE	Changes in general mental functioning	0.no; 1.yes
**r7arthr**		12	0.00107981643120669	8	0.0010372099053546	0	0	TRUE	TRUE	TRUE	TRUE	TRUE	TRUE	TRUE	Other medical history	0.no; 1.yes; 4.disp prev record and no cond; 3.disp prev record and has cond
**r7memrys**		123	0.0110681184198686	75	0.00972384286269934	0	0	TRUE	TRUE	TRUE	TRUE	TRUE	TRUE	TRUE	Memory changes	0.no; 1.yes
**r7dress**		7	0.000629892918203905	6	0.000777907429015947	0	0	TRUE	TRUE	TRUE	TRUE	TRUE	TRUE	TRUE	Problems getting dressed	0.no; 2.can't do; 1.yes; 9.don't do
**r7bath**		11	0.000989831728606137	10	0.00129651238169325	0	0	TRUE	TRUE	TRUE	TRUE	TRUE	TRUE	TRUE	Problems with bathing	0.no; 1.yes; 2.can't do; 9.don't do
**r7toilt**		11	0.000989831728606137	10	0.00129651238169325	0	0	TRUE	TRUE	TRUE	TRUE	TRUE	TRUE	TRUE	Toileting problems	0.no; 1.yes; 9.don't do; 2.can't do
**r7lift**		15	0.00134977053900837	13	0.00168546609620122	2	0.00121802679658952	TRUE	TRUE	TRUE	TRUE	TRUE	TRUE	TRUE	Physical functioning: difficulty lifting 10 pounds	0.no; 2.can't do; 1.yes; 9.don't do
**r7mobila**		16	0.00143975524160893	14	0.00181511733437054	0	0	TRUE	TRUE	TRUE	TRUE	TRUE	TRUE	TRUE	Impaired mobility	
**r7cogtot**		1257	0.113110771168901	941	0.122001815117334	0	0	TRUE	TRUE	TRUE	TRUE	TRUE	TRUE	TRUE	History relevant to cognitive impairment or loss	
**r7fall**	TRUE	22	0.00197966345721227	20	0.00259302476338649	6	0.00365408038976857	TRUE	TRUE	TRUE	TRUE	TRUE	TRUE	TRUE	Falls	
**r7urine**	TRUE	48	0.00431926572482678	37	0.00479709581226501	1	0.000609013398294762	TRUE	TRUE	TRUE	TRUE	TRUE	TRUE	TRUE	Urinary incontinence	
**r7eye**	TRUE	89	0.00800863853144965	80	0.010372099053546	13	0.00791717417783191	TRUE	TRUE	TRUE	TRUE	TRUE	TRUE	TRUE	Sensory problems: Fair or poor eyesight despite use of corrective lenses	
**r7hear**	TRUE	13	0.00116980113380725	9	0.00116686114352392	1	0.000609013398294762	TRUE	TRUE	TRUE	TRUE	TRUE	TRUE	TRUE	Sensory problems: fair or poor hearing despite use of hearing aides	
**r7dizz**	TRUE	38	0.0034194186988212	36	0.00466744457409568	1	0.000609013398294762	TRUE	TRUE	TRUE	TRUE	TRUE	TRUE	TRUE	Physical functioning: Dizziness as persistent problem	
**r7headac**	TRUE	25	0.00224961756501395	22	0.00285232723972514	0	0	TRUE	TRUE	TRUE	TRUE	TRUE	TRUE	TRUE	Headache	
**r7tired**	TRUE	0	0	0	0	0	0	TRUE	TRUE	TRUE	TRUE	TRUE			Tiredness all the time	
**r7gripl**	TRUE	9191	0.827049401601728	6137	0.795669648645145	19	0.0115712545676005	TRUE	TRUE	TRUE	TRUE	TRUE	TRUE	TRUE		
**r7gripr**	TRUE	9204	0.828219202735535	6146	0.796836509788668	29	0.0176613885505481	TRUE	TRUE	TRUE	TRUE	TRUE	TRUE	TRUE		
**r7walkt**	TRUE	9245	0.831908575542158	6183	0.801633605600933	158	0.0962241169305725	TRUE	TRUE	TRUE	TRUE	TRUE	TRUE	TRUE	Slowness: Time to walk 8 ft, converted to time to walk 15 ft. Cutoff criteria according to sex and height remain the same	
**r7muscle**	TRUE	4	0.000359938810402232	3	0.000388953714507974	1	0.000609013398294762	TRUE	TRUE	TRUE	TRUE	TRUE	TRUE	TRUE	Musculoskeletal problems	
**r7memopr**	TRUE	37	0.00332943399622064	11	0.00142616361986257	0	0	TRUE	TRUE	TRUE	TRUE	TRUE			Proxy memory rating	
**r7seizure**	TRUE	0	0	0	0	0	0	TRUE							Seizures, generalized	
**r7grip**		9230	0.830558805003149	6169	0.799818488266563	48	0.0292326431181486	TRUE	TRUE	TRUE	TRUE	TRUE	TRUE	TRUE	Weakness: Grip strength: Weakest 20% (stratified according to sex and BMI)	
**r7wchange**		571	0.0513812651849186	371	0.0481006093608194	37	0.0225334957369062	TRUE	TRUE	TRUE	TRUE	TRUE	TRUE	TRUE	Weight in wave 2002 minus weight in wave 2004 (%)	
**r7underw**		161	0.0144875371186898	113	0.0146505899131337	10	0.00609013398294762	TRUE	TRUE	TRUE	TRUE	TRUE	TRUE	TRUE	Underweight in wave 2004 (%)	
**r7fall_cat2**	TRUE	22	0.00197966345721227	20	0.00259302476338649	6	0.00365408038976857	TRUE	TRUE		TRUE		TRUE		More than 2 falls	
**r7cogimpair**		37	0.00332943399622064	11	0.00142616361986257	0	0	TRUE	TRUE	TRUE	TRUE	TRUE	TRUE	TRUE	Impaired cognition based on performance-based scores or proxy assessment	
**r7frail1_1**		0	0	0	0	0	0	TRUE			TRUE		TRUE		Dizziness as persistent problem, >=2 falls in previous 2 years, or difficulty lifting 10 pounds	
**r7frail1_2**		0	0	0	0	0	0	TRUE			TRUE		TRUE		Weight in wave 2002 minus weight in wave 2004 !10% of weight in wave 2002 or body mass index o18.5 kg/m2	
**r7frail1_3**		0	0	0	0	0	0	TRUE					TRUE		Mild to severe cognitive impairment on performance-based measure or according to proxy and interviewer rating	
**r7frail1_4**		0	0	0	0	0	0	TRUE					TRUE		Fair or poor eyesight despite use of corrective lenses or fair or poor hearing despite use of hearing aides	
**r7actsum**		20	0.00179969405201116	13	0.00168546609620122	4	0.00243605359317905	TRUE	TRUE	TRUE	TRUE	TRUE	TRUE	TRUE	Summary scores of physical activities	
**r7walkt15**	TRUE	9245	0.831908575542158	6183	0.801633605600933	158	0.0962241169305725	TRUE	TRUE	TRUE	TRUE	TRUE	TRUE	TRUE	Time to walk 15 feet	
**r7frail3_2**		6547	0.589129847925853	4321	0.560223000129651	0	0	TRUE	TRUE		TRUE				Yes to either of two CES-D items: (i) Felt that everything I did was an effort in last week. (ii) Could not get going in last week.	
**r7frail3_3**		9471	0.852245118329884	6377	0.826785945805782	0	0	TRUE	TRUE		TRUE				Frequency of three intensities of	

												activity, lowest quintile (stratified according to sex)
**r7frail3_4**		9247	0.832088544947359	6172	0.800207441981071	0	0	TRUE	TRUE	TRUE		Time to walk 8 ft, converted to time to walk 15 ft. Cutoff criteria according to sex and height remain the same
**r7frail3_5**		9471	0.852245118329884	6377	0.826785945805782	0	0	TRUE	TRUE	TRUE	TRUE	Grip strength: Weakest 20% (stratified according to sex and BMI)
**r7fall_cat1**	TRUE	22	0.00197966345721227	20	0.00259302476338649	6	0.00365408038976857	TRUE	TRUE	TRUE	TRUE	More than 1 falls
**r7dresscat**		7	0.000629892918203905	6	0.000777907429015947	0	0	TRUE	TRUE	TRUE	TRUE	Dummy: problem getting dressed
**r7bathcat**		11	0.000989831728606137	10	0.00129651238169325	0	0	TRUE	TRUE	TRUE	TRUE	Dummy: Problems with bathing
**r7toiltcat**		11	0.000989831728606137	10	0.00129651238169325	0	0	TRUE	TRUE	TRUE	TRUE	Dummy: Toileting problems
**r7deprescat**		1248	0.112300908845496	954	0.123687281213536	0	0	TRUE	TRUE	TRUE		Dummy: Feeling sad, blue, depressed
**r7psychcat**		13	0.00116980113380725	9	0.00116686114352392	0	0	TRUE	TRUE	TRUE	TRUE	Dummy: Depression
**r7sleeprcat**		1245	0.112030954737695	954	0.123687281213536	0	0	TRUE	TRUE	TRUE	TRUE	Dummy: Sleep changes
**r7memryscat**		123	0.0110681184198686	75	0.00972384286269934	0	0	TRUE	TRUE	TRUE	TRUE	Dummy: Memory changes
**r7psychscat**		73	0.00656888328984073	48	0.00622325943212758	0	0	TRUE	TRUE	TRUE	TRUE	Dummy: Changes in general mental functioning
**r7strokcat**		6	0.000539908215603347	4	0.000518604952677298	0	0	TRUE	TRUE	TRUE	TRUE	Dummy: Cerebrovascular problems
**r7strokecat**		12	0.00107981643120669	6	0.000777907429015947	0	0	TRUE	TRUE	TRUE	TRUE	Dummy: History of stroke
**r7diabscat**		79	0.00710879150544407	50	0.00648256190846623	0	0	TRUE	TRUE	TRUE	TRUE	Dummy: History of diabetes mellitus
**r7heartcat**	TRUE	15	0.00134977053900837	14	0.00181511733437054	1	0.000609013398294762	TRUE	TRUE	TRUE	TRUE	Dummy: Cardiac problems
**r7lungcat**		10	0.000899847026005579	8	0.0010372099053546	1	0.000609013398294762	TRUE	TRUE	TRUE	TRUE	Dummy: Lung problems
**r7cancrcat**		10	0.000899847026005579	5	0.000648256190846623	0	0	TRUE	TRUE	TRUE	TRUE	Dummy: Malignant disease
**r7arthrcat**		12	0.00107981643120669	8	0.0010372099053546	0	0	TRUE	TRUE	TRUE	TRUE	Dummy: Other medical history

Using syndrome mining with the Health and Retirement Study to identify the deadliest and least deadly frailty syndromes

Authors

Yi-Sheng Chao, Chao-Jung Wu, Hsing-Chien Wu, Hui-Ting Hsu, Lien-Cheng Tsao, Yen-Po Cheng, Yi-Chun Lai, Wei-Chih Chen, MD

Supplemental material 3

The relationships between input symptoms and mined syndromes.

Figure 1. The frequencies of constituting significant PC-based syndromes and the input symptom p values for mortality prediction

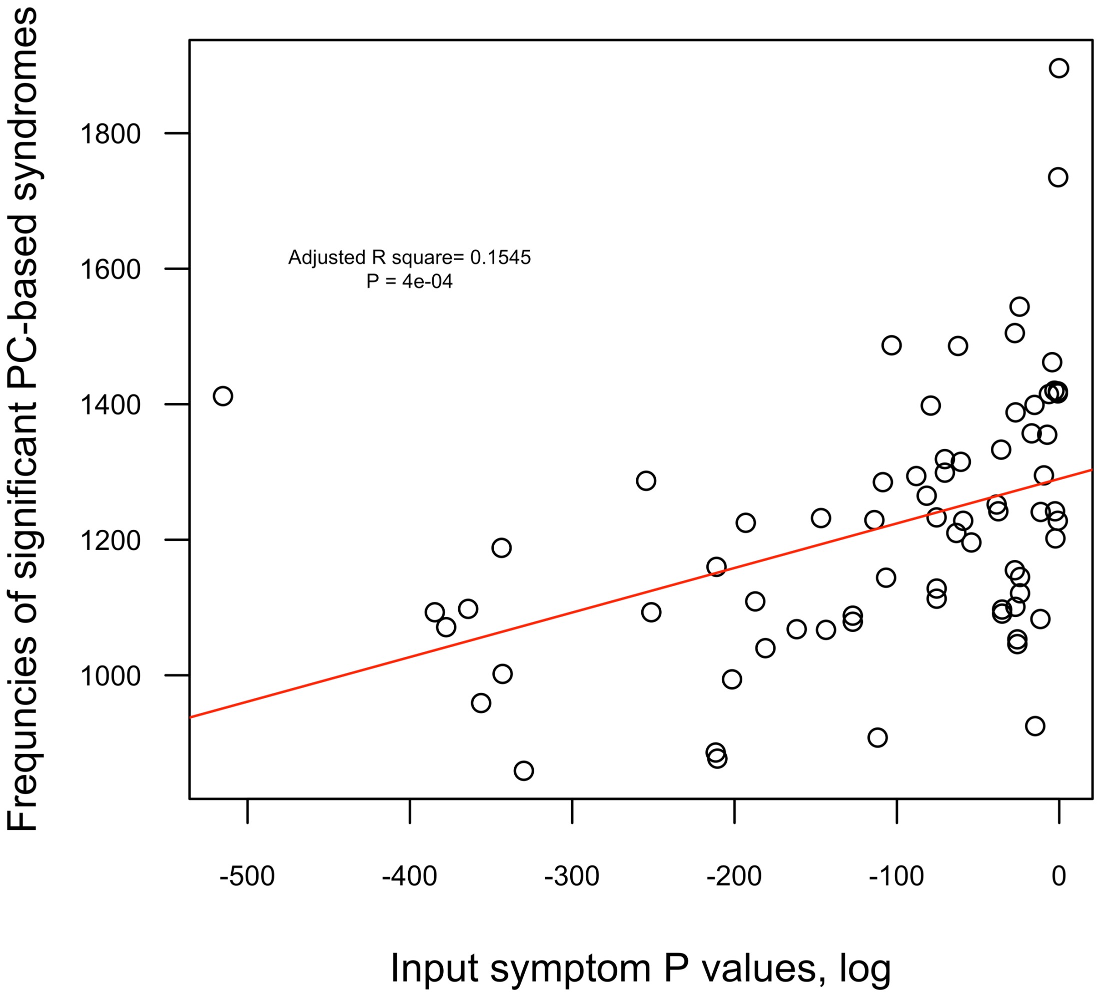

Figure 2. The frequencies of constituting significant 4-item equal-weight syndromes and the input symptom p values for mortality prediction

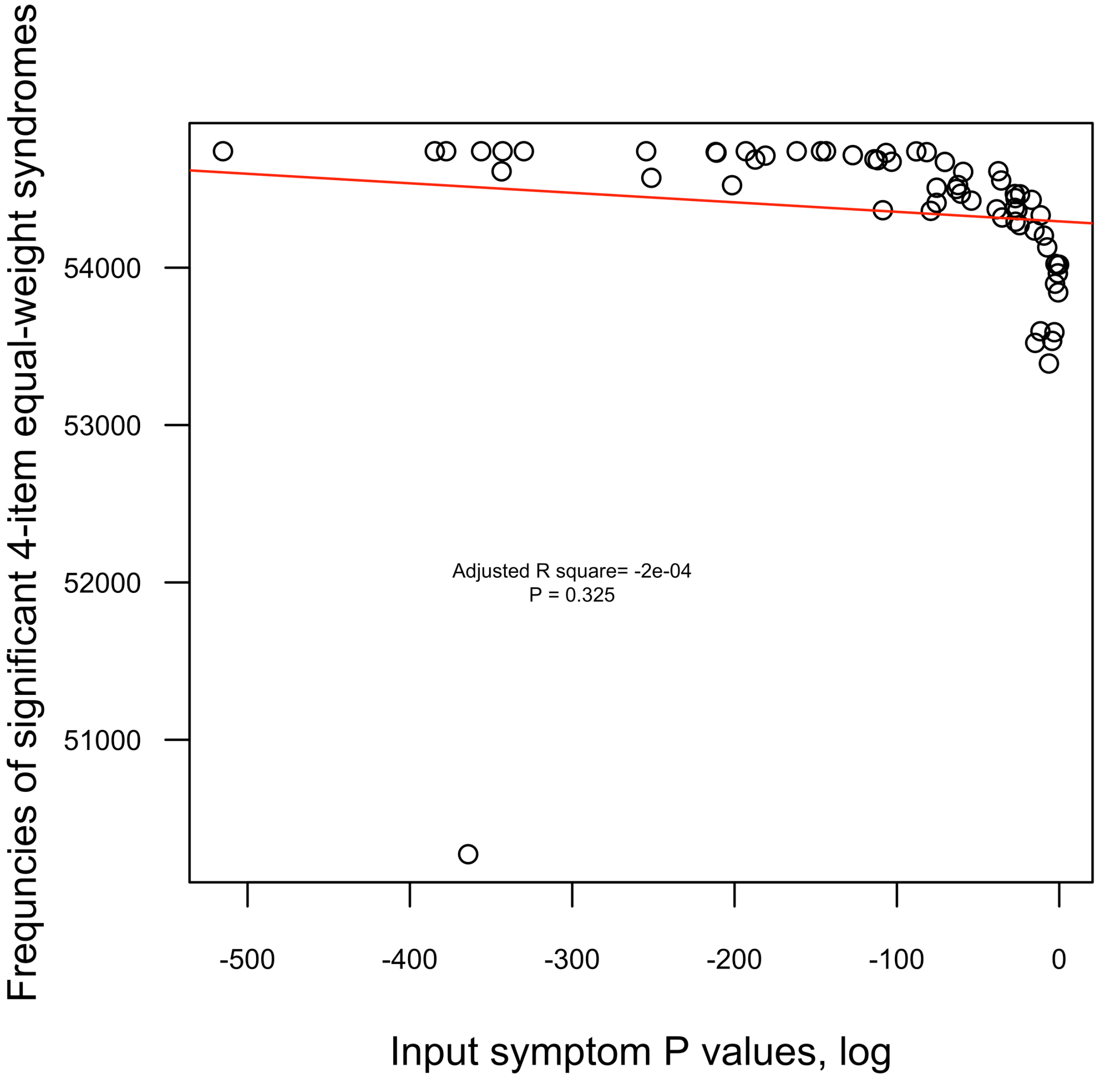

Figure 3. The frequencies of constituting significant deadly PC-based syndromes and the input symptom p values for mortality prediction

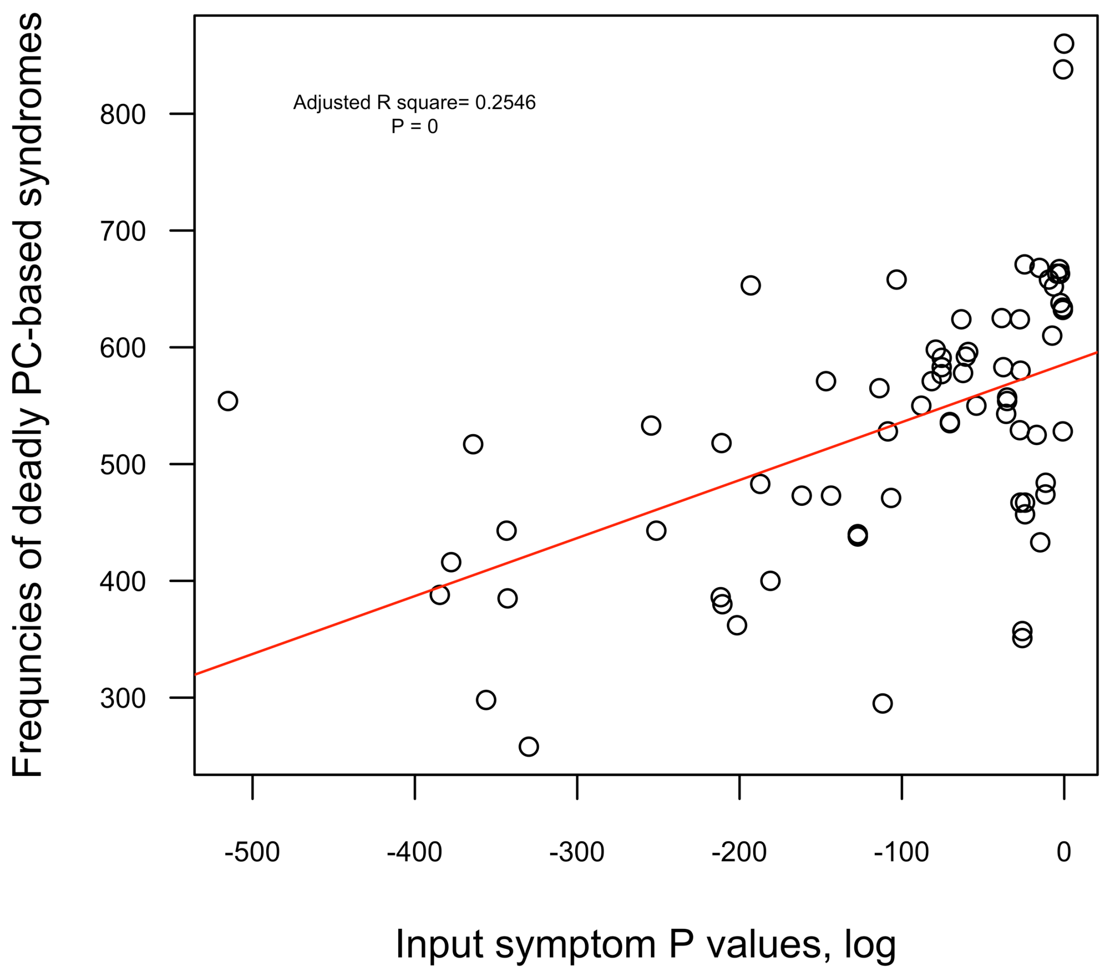

Figure 4. The frequencies of constituting significant deadly 4-item equal-weight syndromes and the input symptom p values for mortality prediction

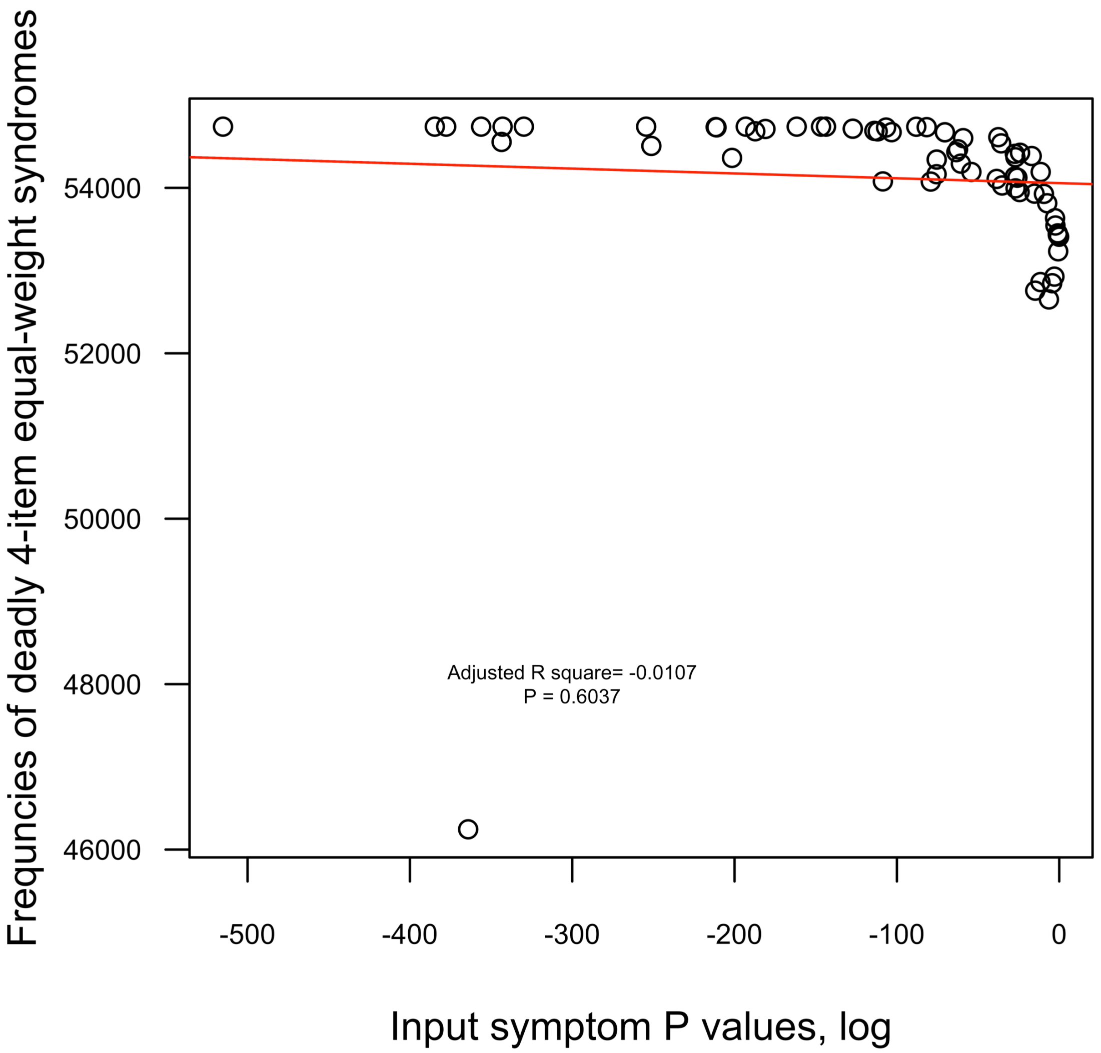

Figure 5. The frequencies of constituting significant death-averse PC-based syndromes and the input symptom p values for mortality prediction

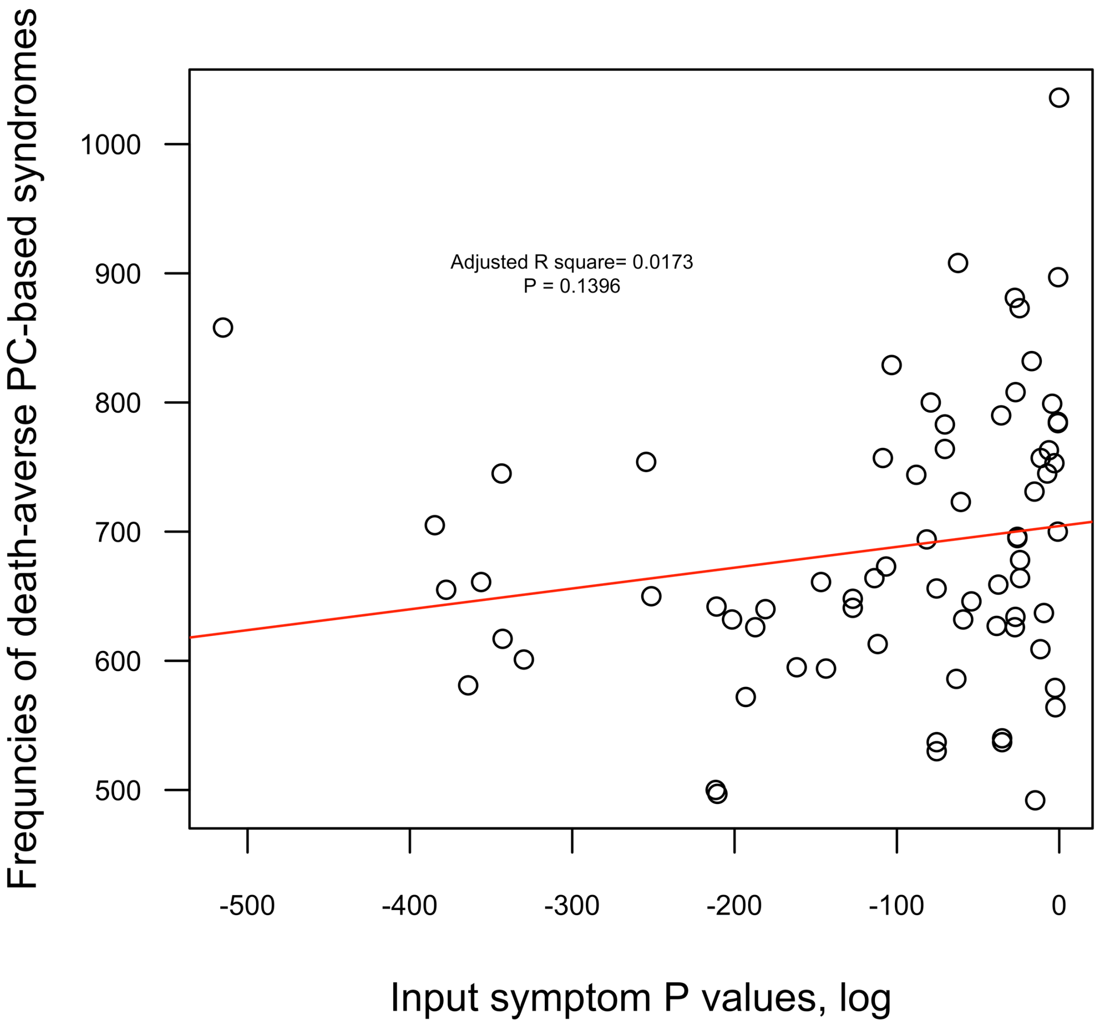

Figure 6. The frequencies of constituting significant death-averse 4-item equal-weight syndromes and the input symptom p values for mortality prediction

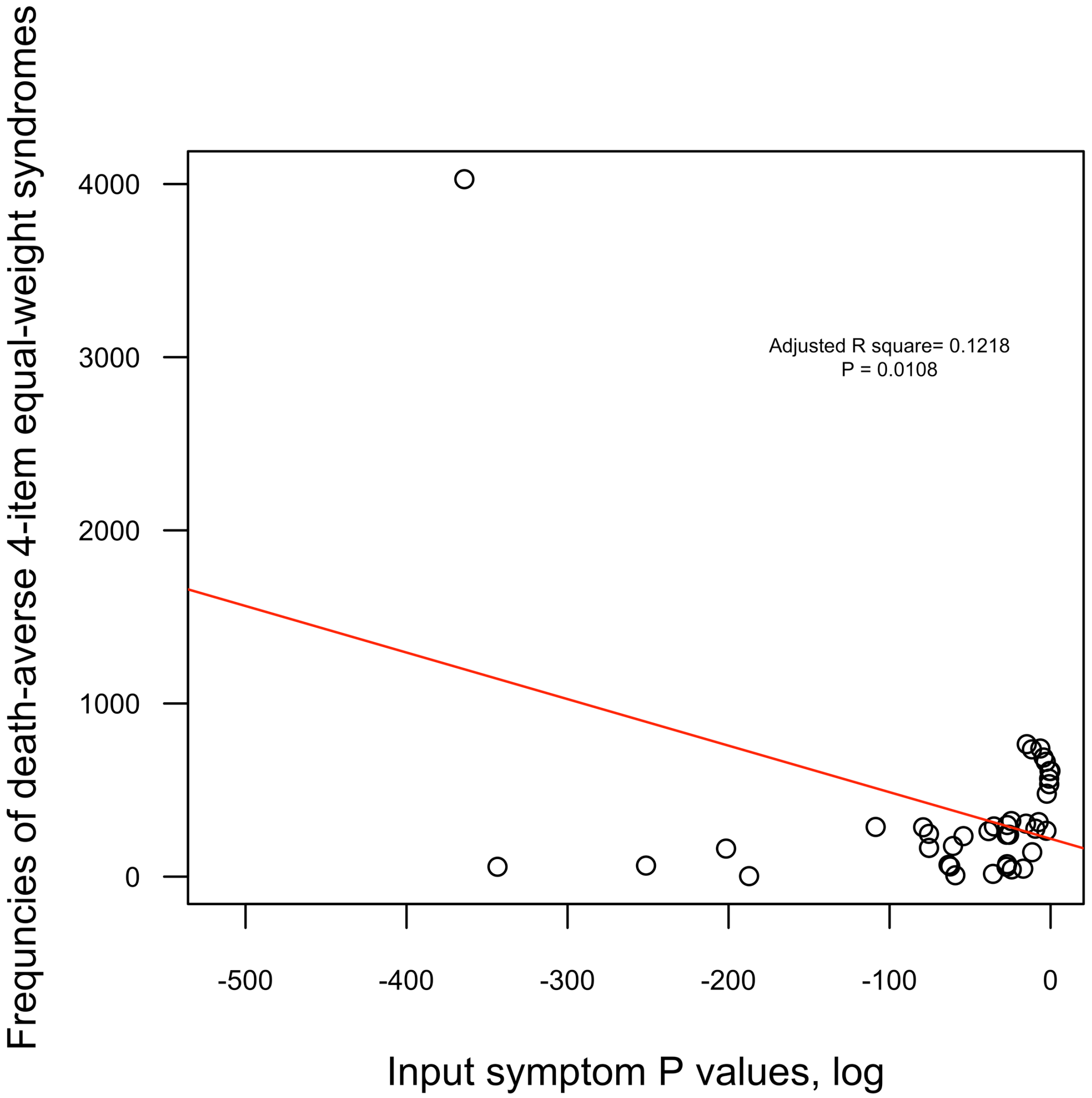

Figure 7. The frequencies of constituting significant PC-based syndromes and the input symptom variances

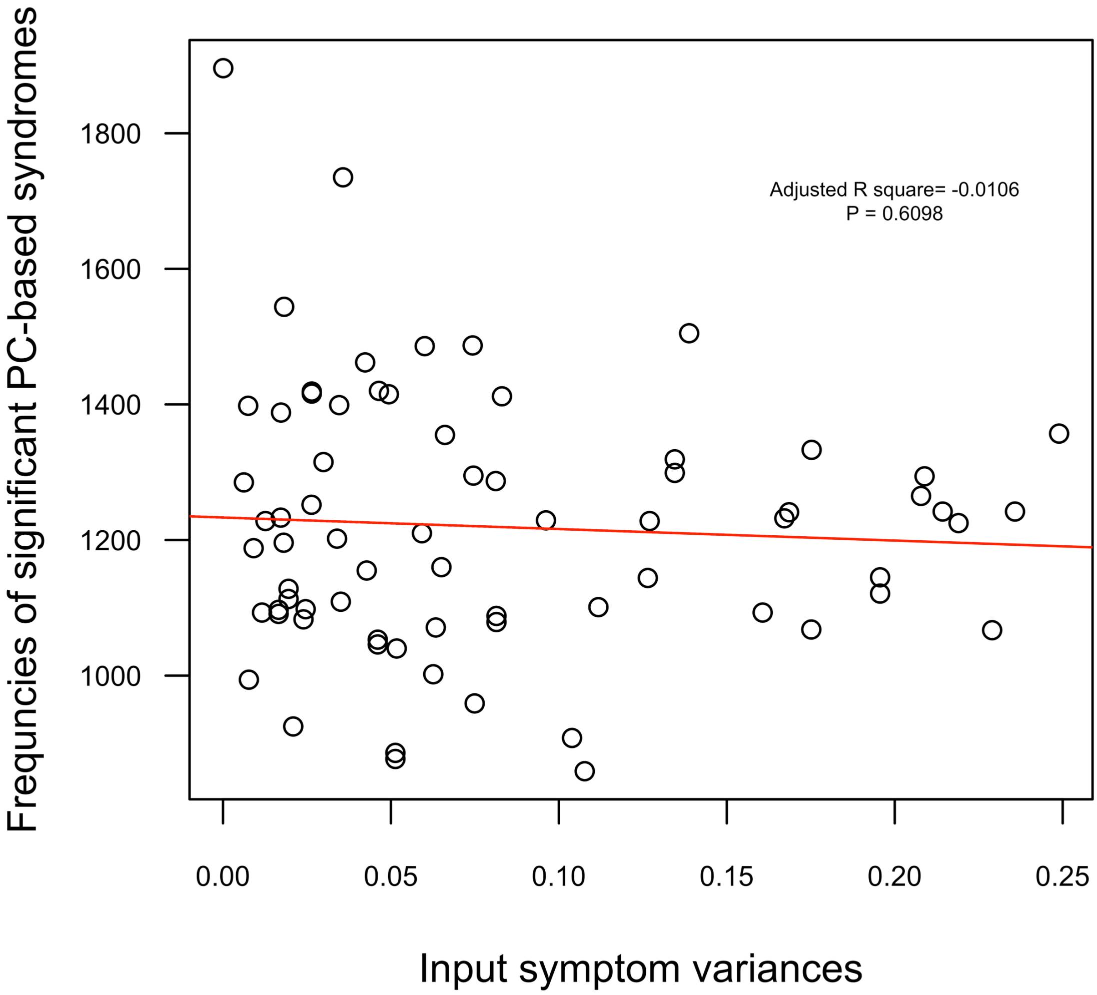

Figure 8. The frequencies of constituting significant 4-item equal-weight syndromes and the input symptom variances

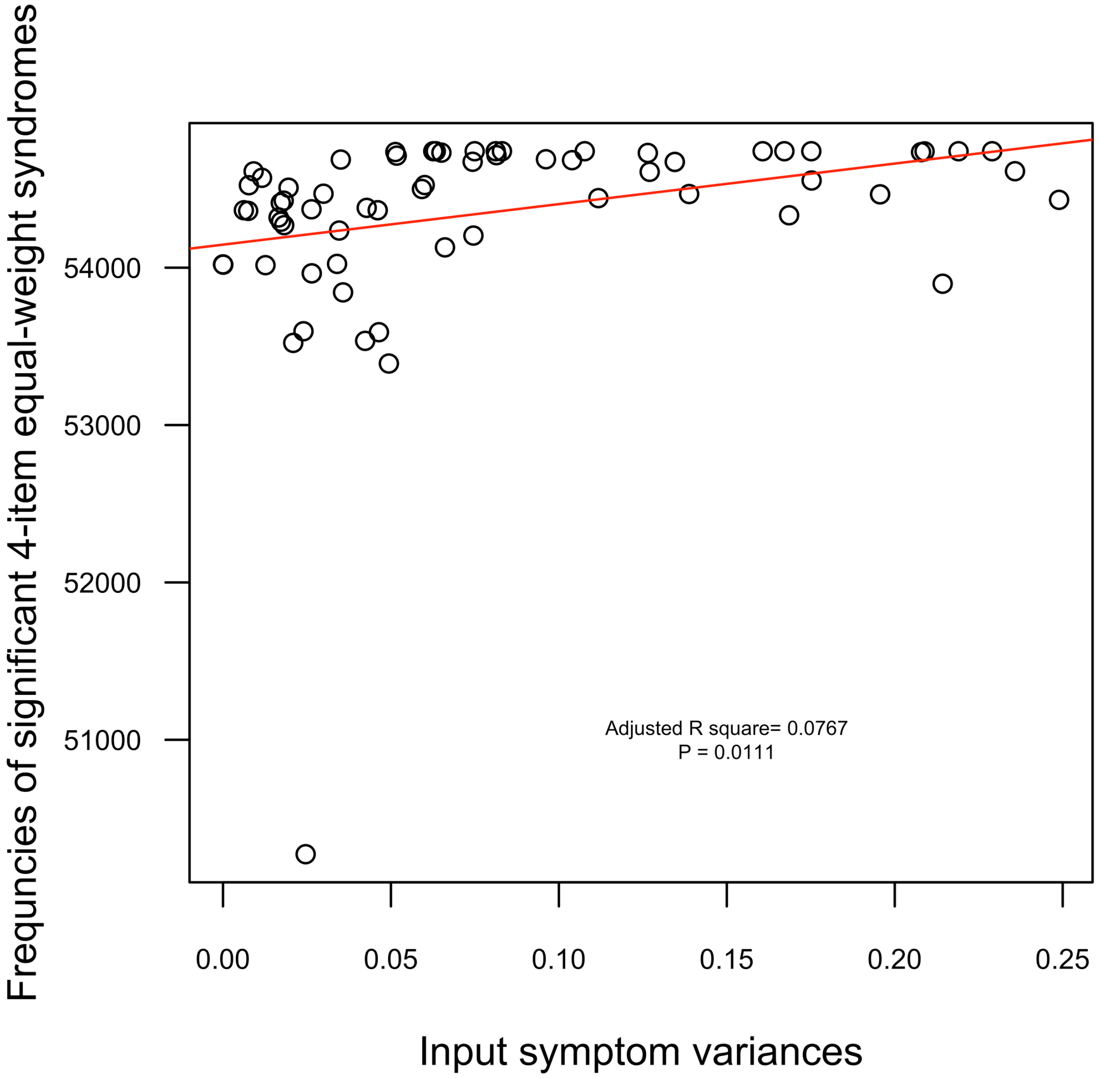

Figure 9. The frequencies of constituting significant deadly PC-based syndromes and the input symptom variances

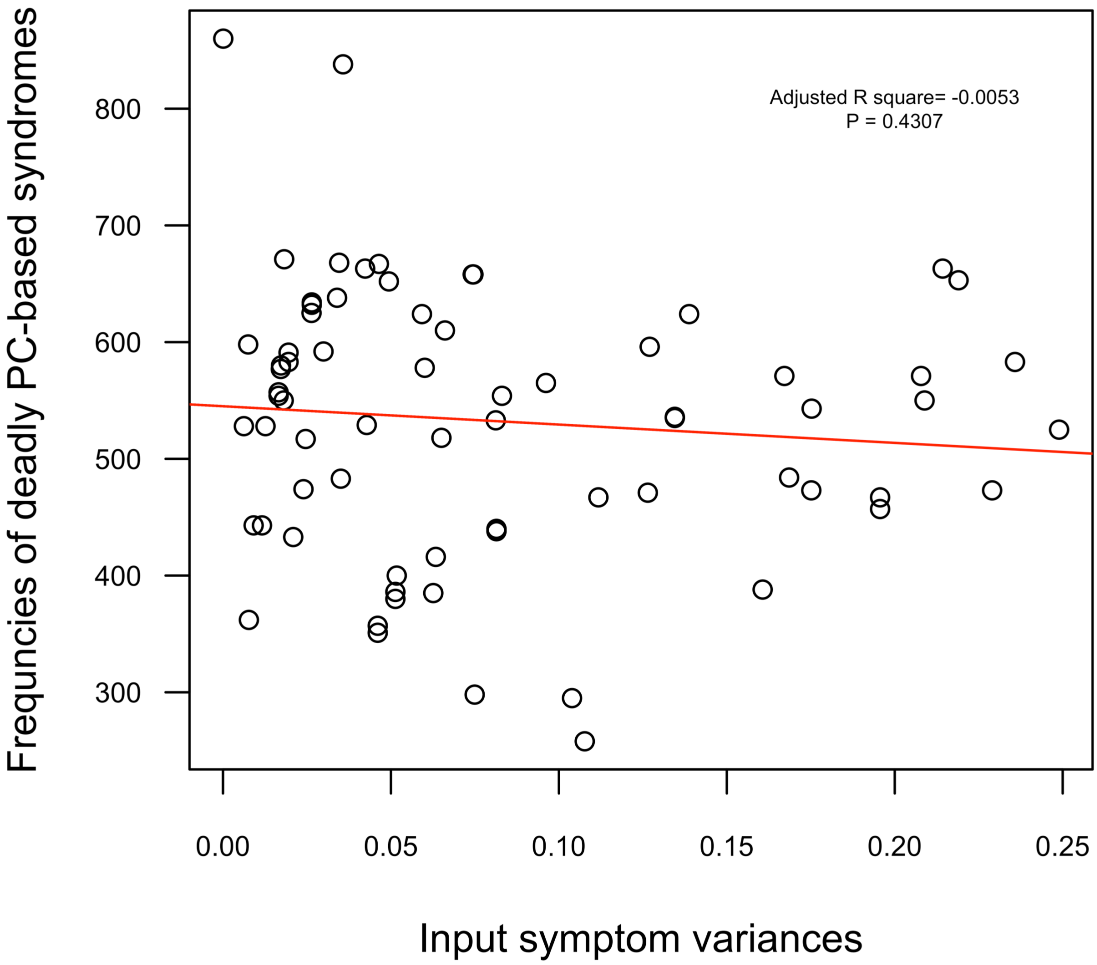

Figure 10. The frequencies of constituting significant deadly 4-item equal-weight syndromes and the input symptom variances

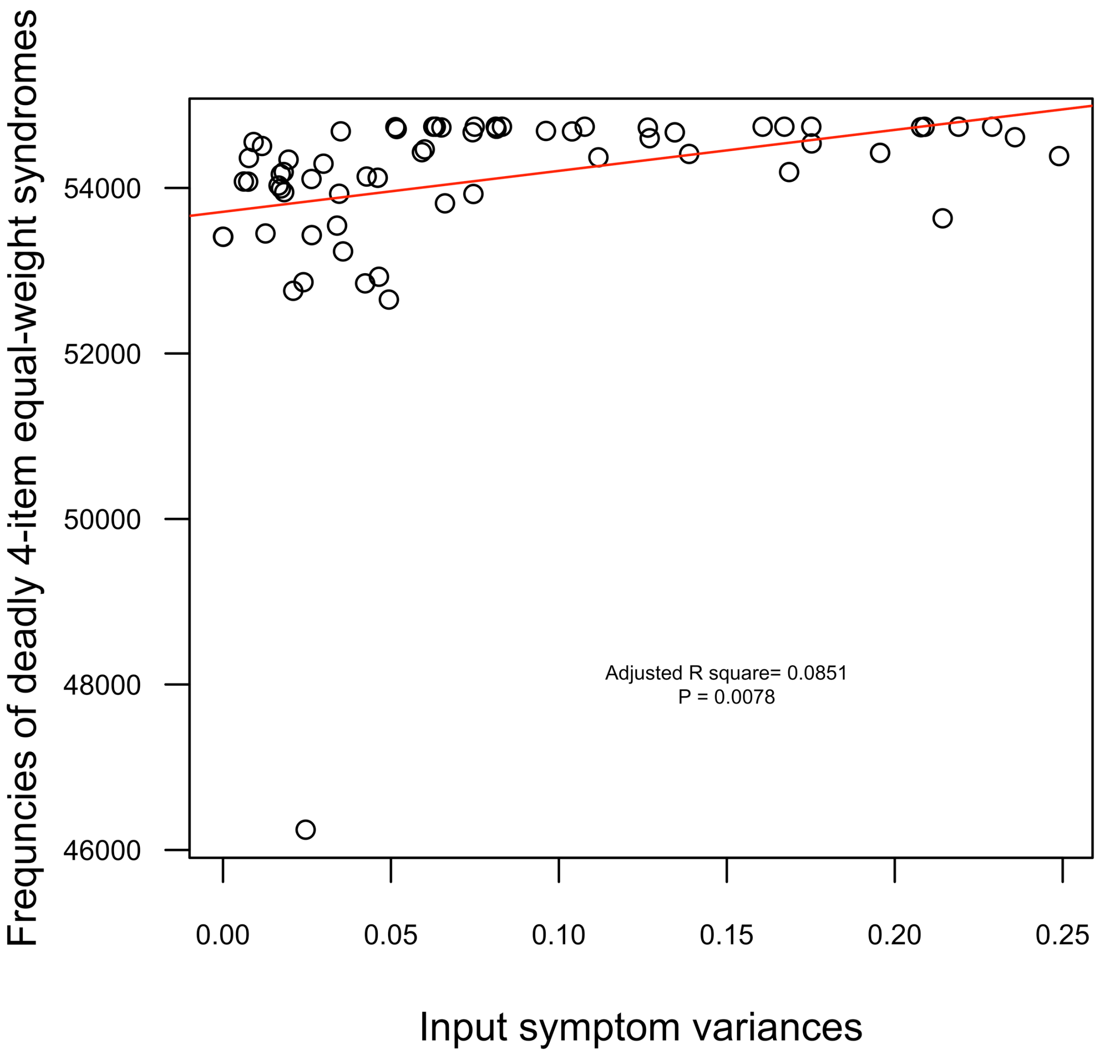

Figure 11. The frequencies of constituting significant death-averse PC-based syndromes and the input symptom variances

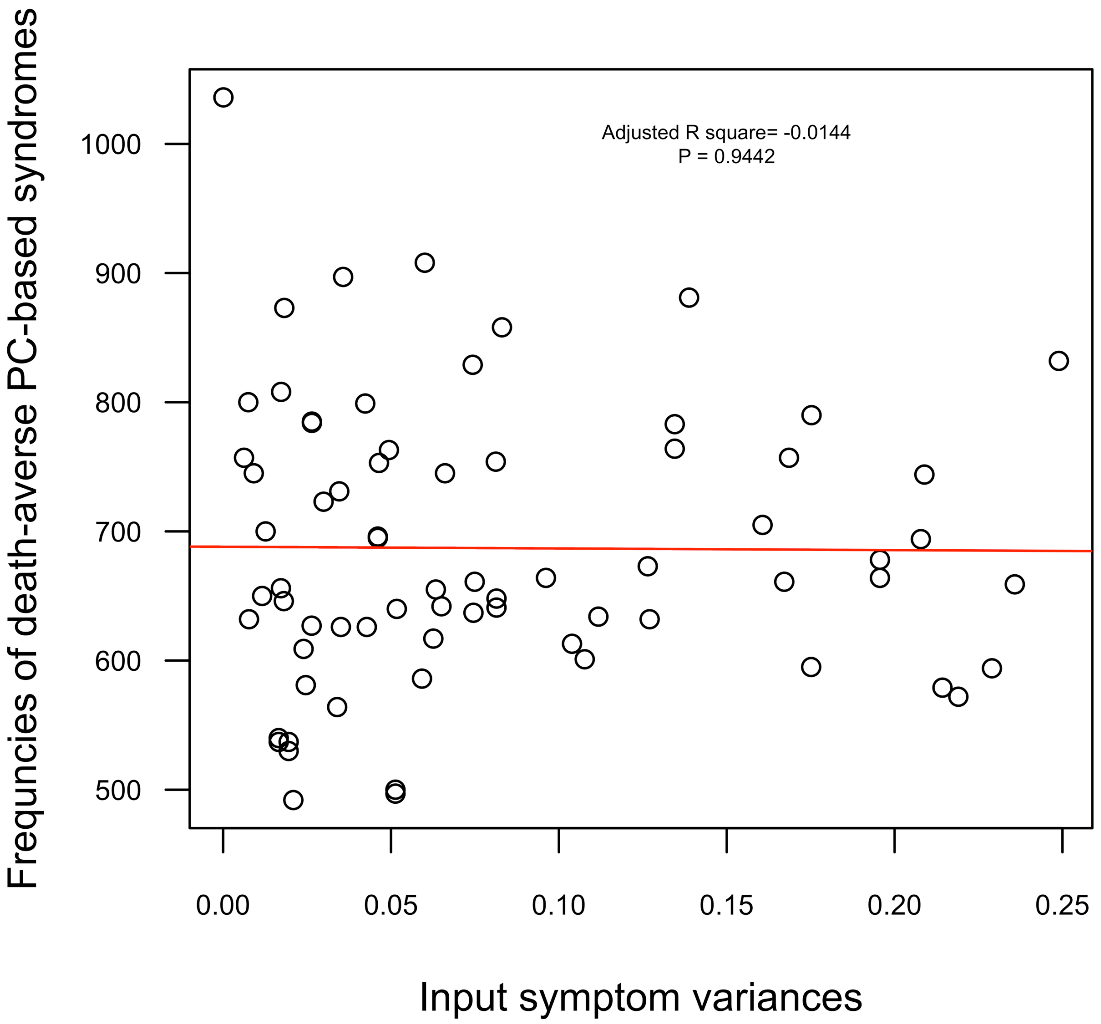

Figure 12. The frequencies of constituting significant death-averse 4-item equal-weight syndromes and the input symptom variances

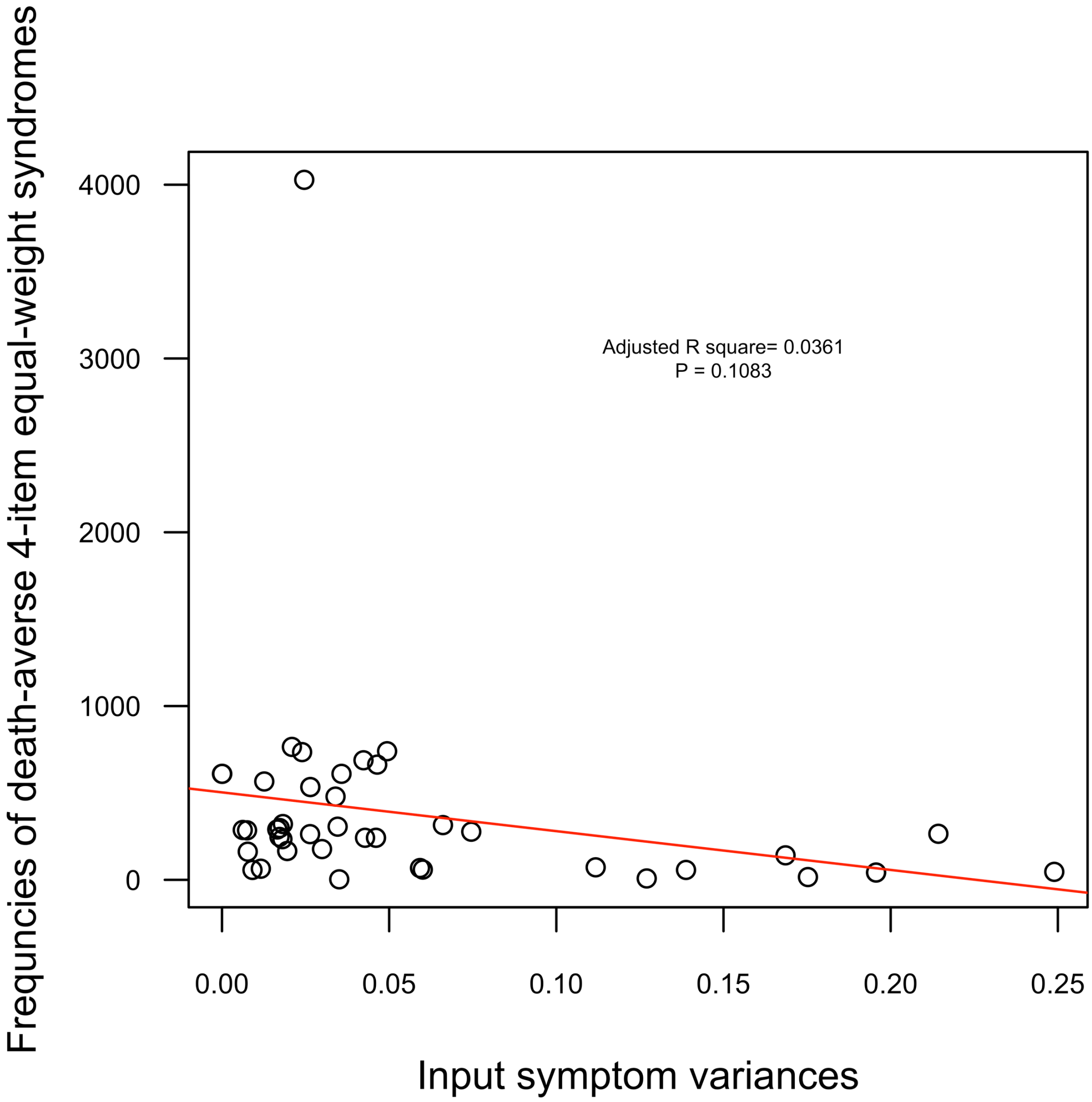

Figure 13. The frequencies of constituting significant PC-based syndromes and the input symptom regression coefficients for mortality prediction

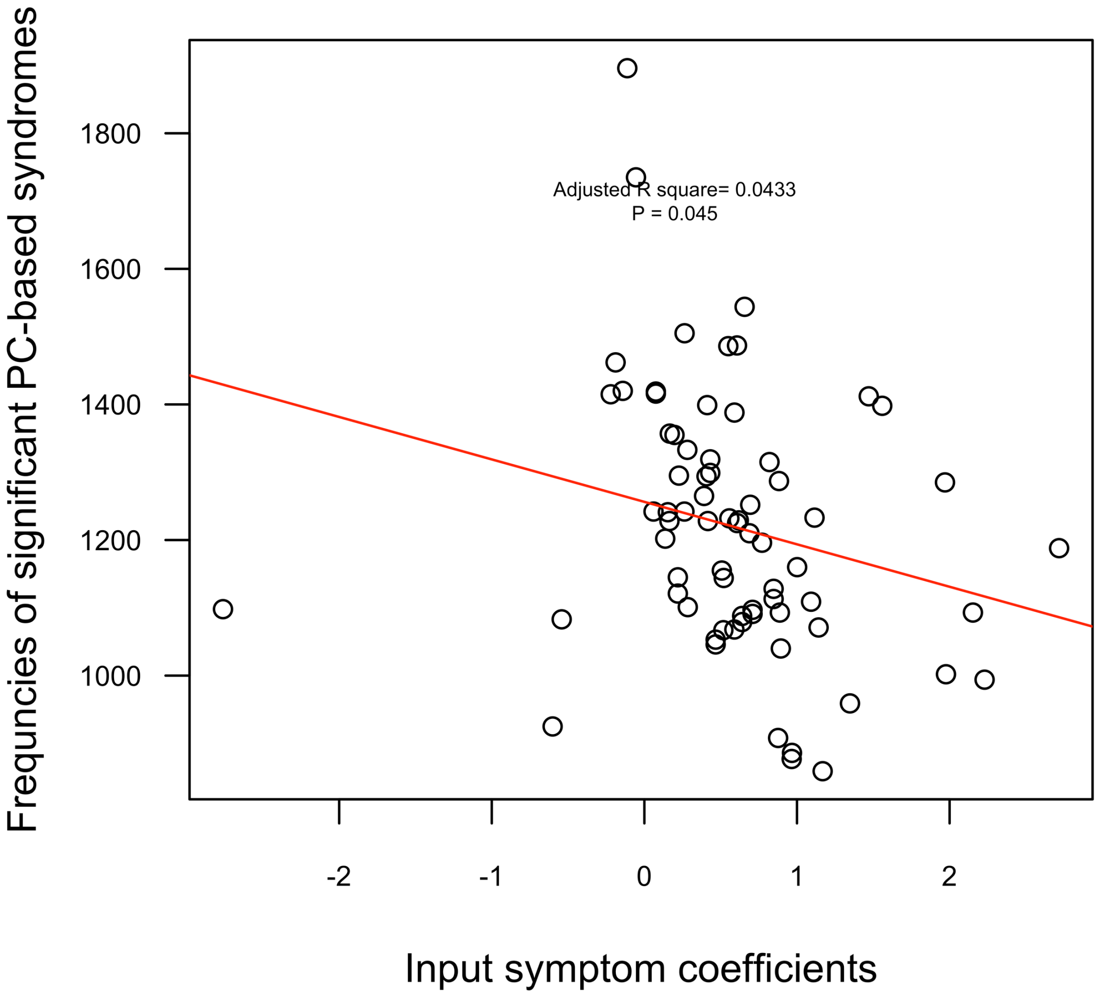

Figure 14. The frequencies of constituting significant 4-item equal-weight syndromes and the input symptom regression coefficients for mortality prediction

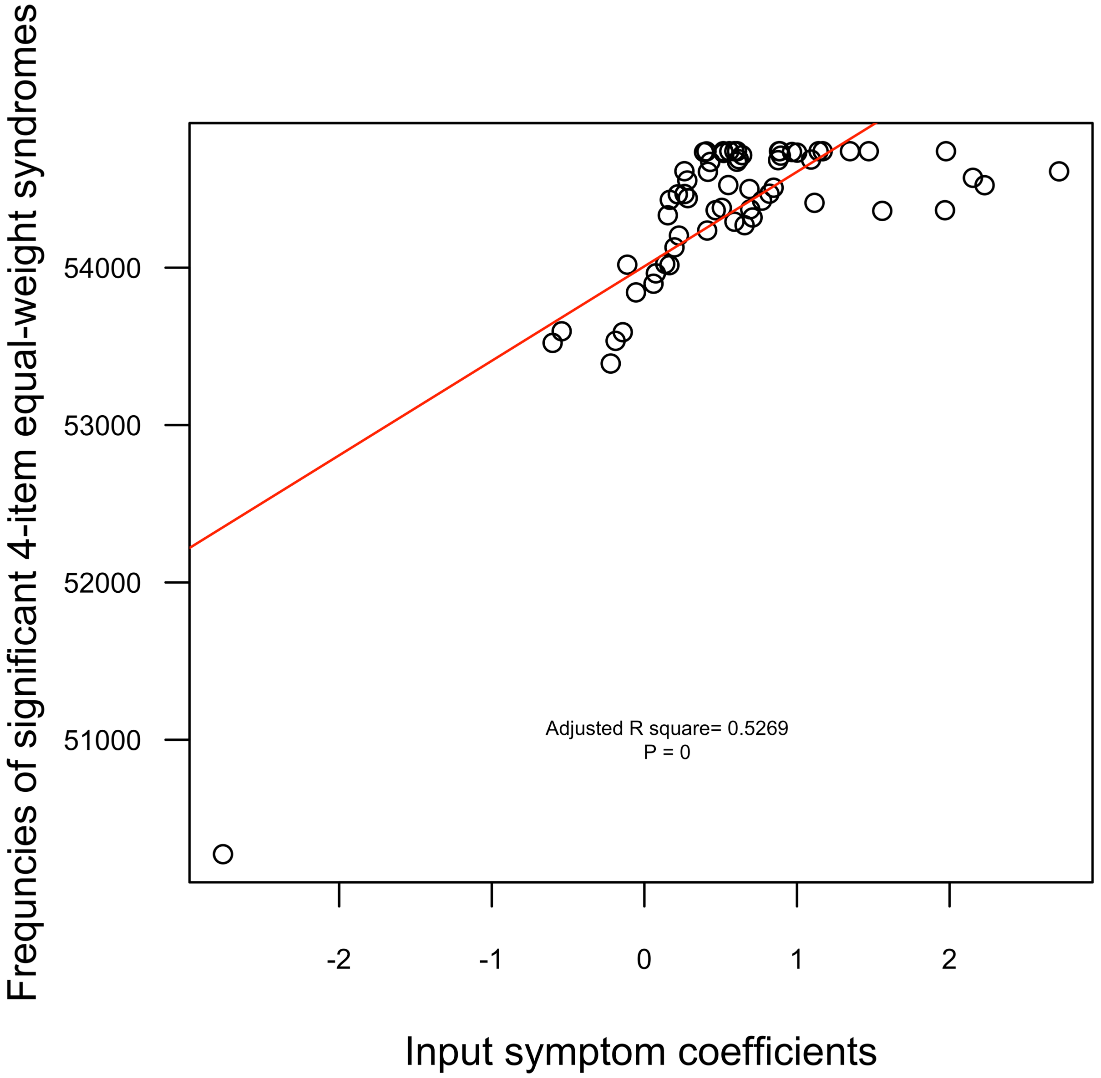

Figure 15. The frequencies of constituting significant deadly PC-based syndromes and the input symptom regression coefficients for mortality prediction

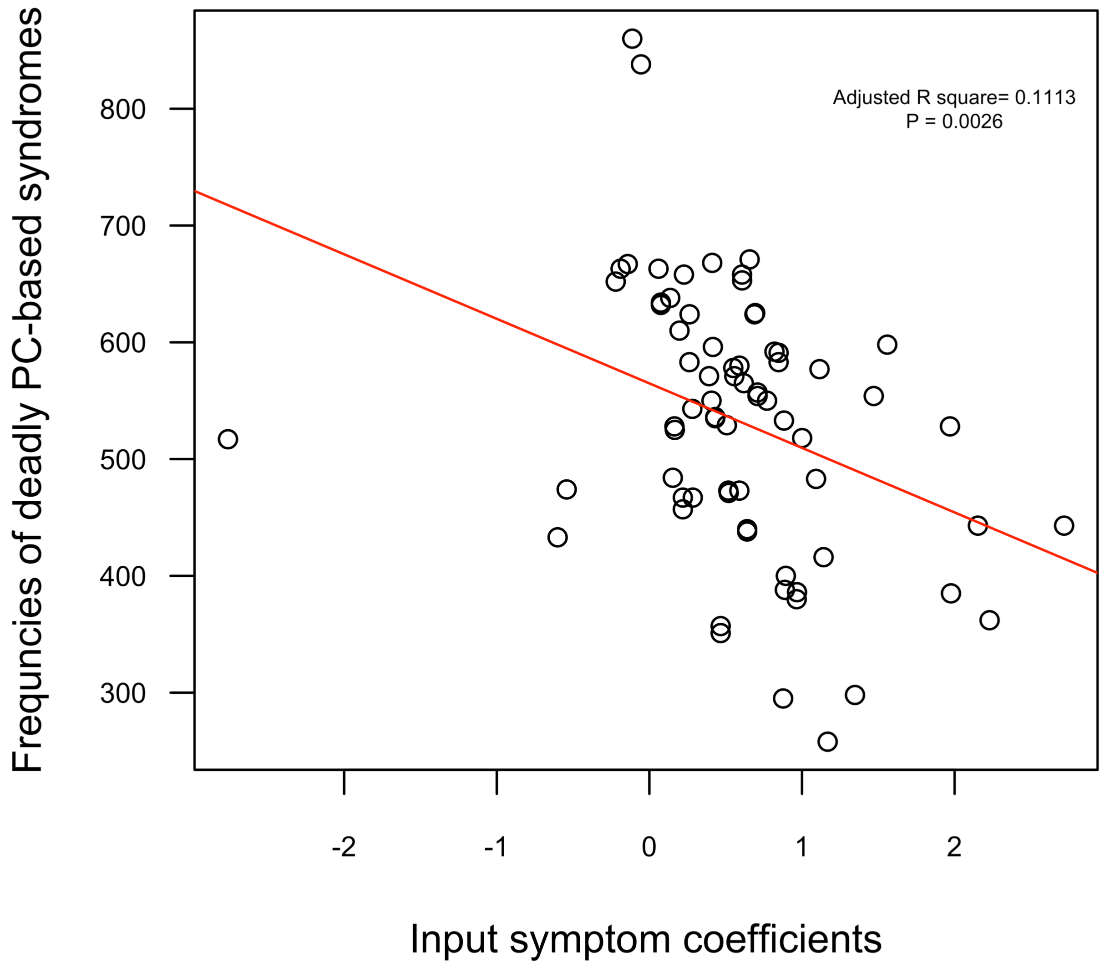

Figure 16. The frequencies of constituting significant deadly 4-item equal-weight syndromes and the input symptom regression coefficients for mortality prediction

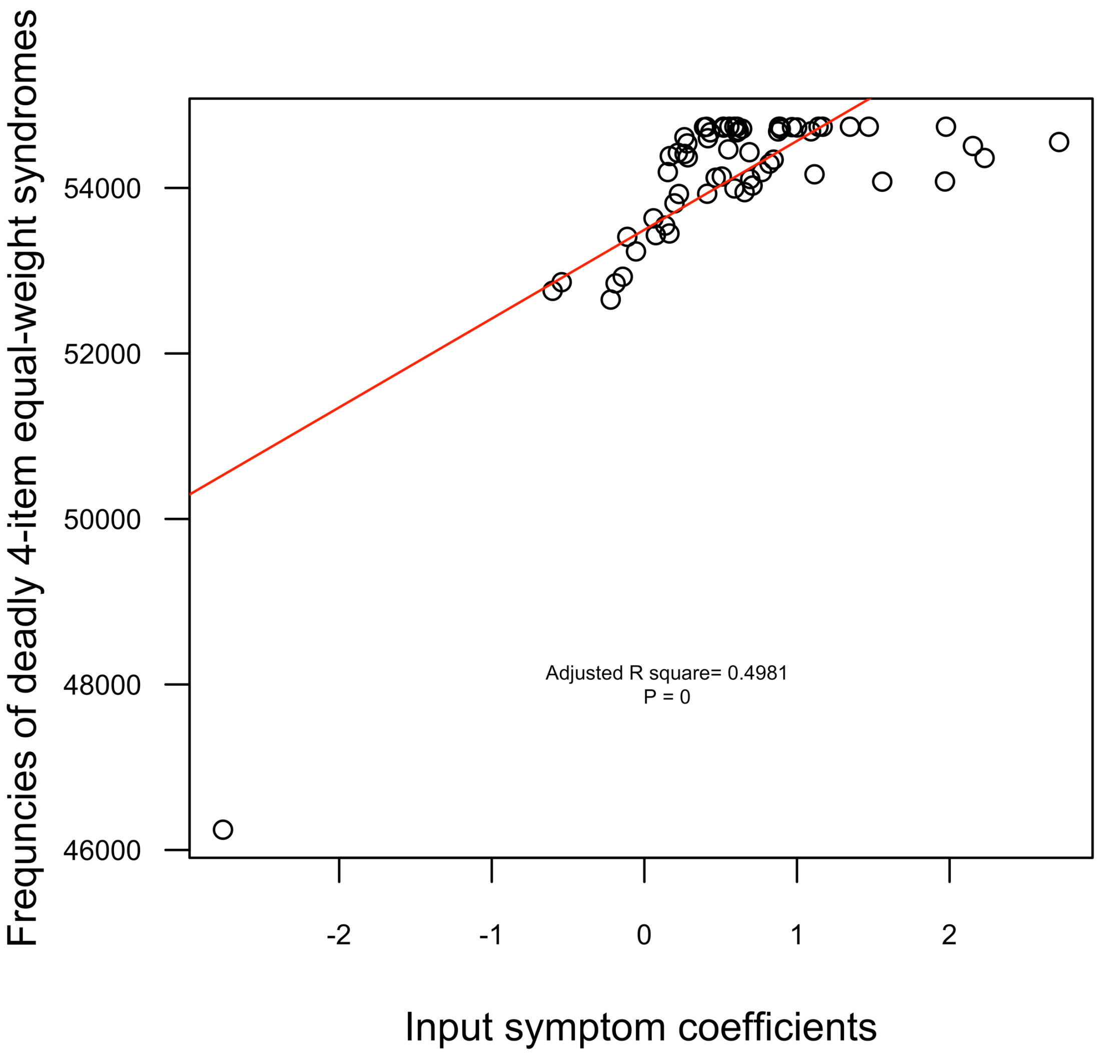

Figure 17. The frequencies of constituting significant death-averse PC-based syndromes and the input symptom regression coefficients for mortality prediction

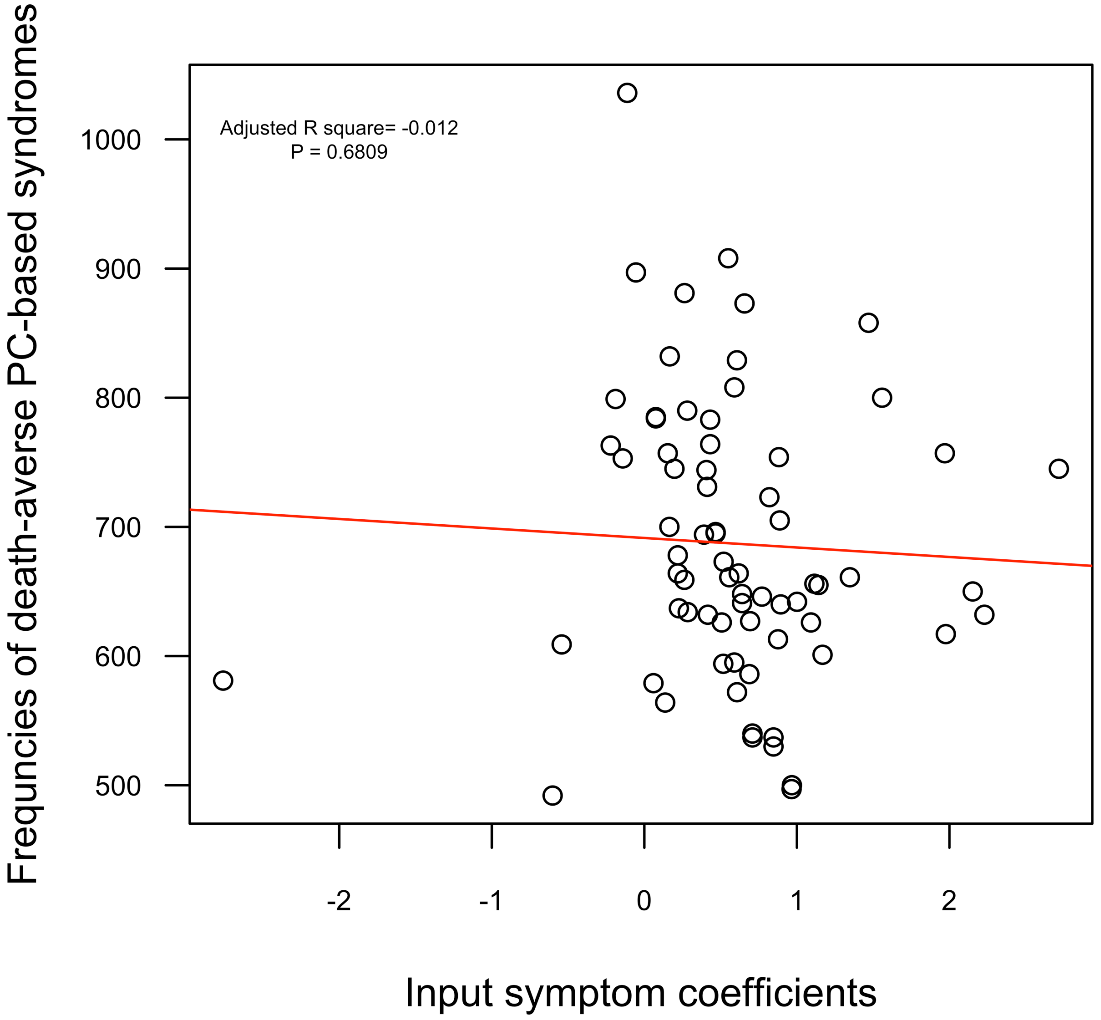

Figure 18. The frequencies of constituting significant death-averse 4-item equal-weight syndromes and the input symptom regression coefficients for mortality prediction

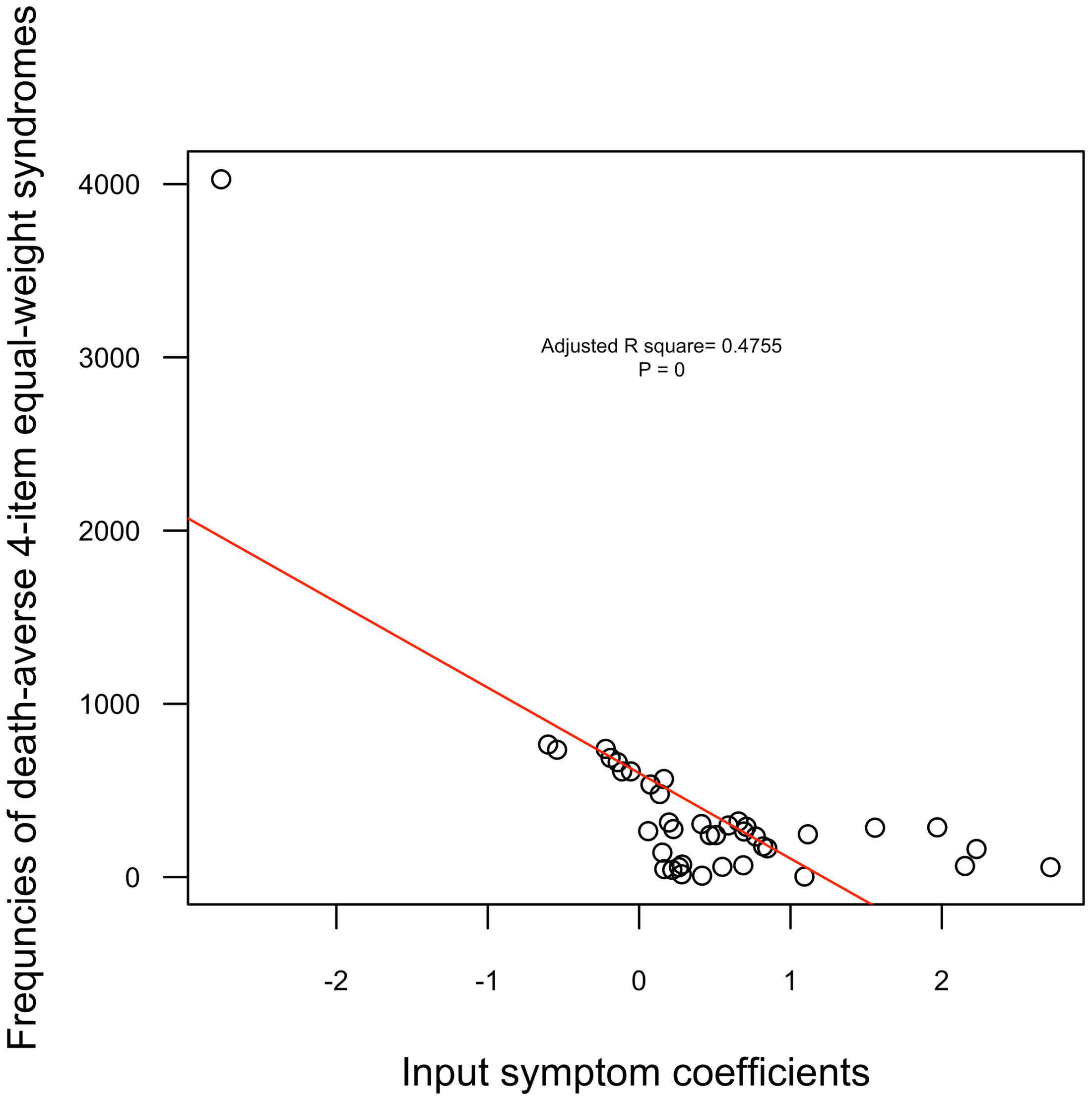

Supplement: Supplementary file 1 — Supplemental materials. [file 41598_2020_60869_MOESM1_ESM.pdf]
